# Cross-sectional and longitudinal associations of domain-specific physical activity composition with health-related quality of life in childhood and adolescence in Australia

**DOI:** 10.1186/s12966-023-01466-6

**Published:** 2023-06-06

**Authors:** Byron J. Kemp, Dorothea Dumuid, Kar Hau Chong, Anne-Maree Parrish, Dylan Cliff

**Affiliations:** 1 Early Start, University of Wollongong, Northfields Ave, Wollongong, NSW 2522 Australia; 2 School of Education, Faculty of the Arts, Social Sciences and Humanities, University of Wollongong, Northfields Ave, Wollongong, NSW 2522 Australia; 3 Alliance for Research in Exercise, Nutrition and Activity (ARENA), Allied Health & Human Performance, University of South Australia, Cnr North Terrace & Frome Rd, Adelaide, SA 5001 Australia; 4 School of Health and Society, Faculty of the Arts, Social Sciences and Humanities, University of Wollongong, Northfields Ave, Wollongong, NSW 2522 Australia; 5 Illawarra Health and Medical Research Institute, University of Wollongong, Northfields Ave, Wollongong, NSW 2522 Australia

**Keywords:** Organized sport, Non-organized physical activity, Active play, Active transport, Occupational physical activity, Household physical activity, Children, Adolescents, Compositional data analysis, Public health

## Abstract

**Background:**

Health benefits have been linked with physical activity (PA), as well as some domains of PA among youth (e.g. organized PA and active transport). However, less is known about whether some PA domains are more beneficial than others. There is also a lack of evidence about whether health outcomes are related to the composition of PA (i.e. the share of PA spent in different domains). This study aimed to identify: (1) how the absolute durations of organized PA, non-organized PA, active transport and active chores/work at 10-11y are individually associated with physical, psychosocial and total health-related quality of life (HRQOL) at 10-11y and 12-13y; and (2) how the domain-specific composition of PA at 10-11y is associated with HRQOL at 10-11y and 12-13y.

**Methods:**

Data from the Longitudinal Study of Australian Children were used in cross-sectional (n ≥ 2730) and longitudinal analyses (n ≥ 2376). Measurement included the Pediatric Quality of Life Inventory (PedsQL™) for HRQOL domains and one-day time-use diaries (TUDs) for PA domains. Robust linear regression models were used, controlling for age, sex, pubertal status, socioeconomic position, body mass index and TUD context (season and school attendance). Compositional models additionally adjusted for total PA duration and longitudinal models controlled for baseline PedsQL™ scores.

**Results:**

Non-compositional models indicated that the duration of organized PA, and to a lesser extent non-organized PA, were positively but weakly associated with some HRQOL outcomes at 10-11y. These trends were not reflected in longitudinal models, although a 30-min increase in non-organized PA per day did predict marginally better psychosocial HRQOL at 12-13y (+ 0.17%; 95%CI =  + 0.03%, + 0.32%). Compositional models revealed that a 30-min increase in organized PA relative to other domains was positively but weakly associated with physical (+ 0.32%; 95%CI =  + 0.01%, + 0.63%), psychosocial (+ 0.41%; 95%CI =  + 0.11%, + 0.72%) and total HRQOL (+ 0.39%; 95%CI =  + 0.12%, + 0.66%) at 10-11y. However, the overall PA composition at 10-11y was not related to HRQOL at 12-13y.

**Conclusions:**

Non-compositional and compositional models generally concurred on the direction of cross-sectional and longitudinal relationships (and lack thereof) between PA domains and HRQOL outcomes. The strongest associations were cross-sectional between organized PA and HRQOL at 10-11y. However, all associations between PA domains and HRQOL outcomes were weak and may not be clinically meaningful.

**Supplementary Information:**

The online version contains supplementary material available at 10.1186/s12966-023-01466-6.

## Background

Physical activity (PA) participation has been beneficially linked with a number of health outcomes (e.g., adiposity, cardiometabolic biomarkers and physical fitness) during childhood and adolescence [1]. However, PA participation has been frequently reported to be insufficient among youth and is prone to decline with age [2]. As a result, PA promotion is a key public health priority among researchers and policymakers [3].

An emerging area of research relates to particular groupings of PA participation, known as domains of PA [4]. Common PA domains include transportation, active chores/household work and leisure-time PA [5]. Active transport includes activities such as walking/cycling to school or other locations [6]. Active chores/household work may include activities such as cleaning and gardening [7, 8]. Leisure-time PA is sometimes divided into organized and non-organized activities among children and adolescents. Organized PA includes structured activities that usually have rules and are led by an adult (e.g. club sports or dance classes) [9]; and non-organized PA includes more unstructured, freely chosen and self-directed activities (e.g. playground games and kicking a ball in a park) [6]. Studies of PA domains such as these are useful because they can provide a more nuanced understanding of different PA participation patterns. For example, evidence from a systematic review suggests that participation in active transport may be likely to increase during childhood [10] and a previous Australian longitudinal study identified that non-organized PA may be particularly prone to decline between childhood and adolescence [11].

One aspect of this field of research relates to the health benefits associated with participation in domains of PA [4]. Both physical and psychosocial outcomes are important to consider, as PA context might affect these differently. Previous reviews have reported physical and psychosocial outcomes as being associated with organized PA (e.g. [12, 13]), non-organized PA (e.g. [14, 15]) and active transport (e.g. [16, 17]). By contrast, very few studies have tested health associations for active chores/work, and no known studies have concurrently investigated longitudinal associations between the four aforementioned PA domains and a health-related outcome. Some studies have investigated associations between three of these aforementioned PA domains (i.e., organized PA, non-organized PA, active transport) and health-related outcomes, although these studies were all cross-sectional in nature [18-22]. Several other studies have explored health-related associations for two of the aforementioned PA domains (i.e., organized PA, active transport), with most adopting cross-sectional methods [23-26], although some studies were longitudinal [27-29]. However to the authors’ knowledge, no existing studies have explored longitudinal associations between health-related outcomes and the duration of organized PA, non-organized PA, active transport and active chores/work in the same sample of youth.

Health-related quality of life (HRQOL) is one outcome that is often used as a global measure of individuals’ perceptions of their own health and well-being [30]. Generally, HRQOL is associated with higher PA levels among children, however few studies have considered whether the domain of PA is important in this relationship [30]. Five studies have reported positive associations between binary (Yes vs No) participation in organized PA/sports and HRQOL [31-35]. Two studies reported weak beneficial associations between the duration of organized PA/sport and HRQOL [36, 37]. A third study reported that organized PA was positively associated with HRQOL and that active transport was negatively associated with HRQOL among girls [19]. Finally, one study reported that overall HRQOL was weakly associated with organized PA but not non-organized PA [38]. Importantly, most of these previous studies were cross-sectional [19, 31-33, 36, 38], while those with longitudinal designs only tested associations for a single domain in isolation (organized PA or sports) [34, 35, 37]. By contrast, testing longitudinal associations for multiple PA domains concurrently would provide useful information about whether certain PA domains are more strongly associated with future HRQOL outcomes than others.

Another way to explore the potential associations between domain-specific PA and HRQOL may be to consider the *composition* of PA. Compositional approaches have been previously used to analyse constrained data that add to a fixed total value, such as 24-h movement behaviours (i.e. PA, sleep and sedentary behaviour) [39, 40]. However, it is also possible to apply compositional approaches in situations where the total value varies between individuals, such as accelerometer data collected using a waking wear protocol [41]. The CoDA-with-a-total (or tCoDA) approach, which enables the inclusion of all parts of a composition while taking into account the total value, has previously been used to explore the associations between body composition/mass and health outcomes [42, 43]. To our knowledge, the CoDA-with-a-total approach has not yet been applied to domain-specific PA and represents a novel way to explore this topic. A key outcome of this approach would be to identify whether the share of PA time in different domains is important for health. It could also enable a comparison of whether health-related outcomes are mainly associated with the composition of PA or the absolute duration of domain-specific PA (or both). Therefore, this study aimed to identify: (1) how the absolute durations of organized PA, non-organized PA, active transport and active chores/work at 10-11y are individually associated with physical, psychosocial and total HRQOL at 10-11y and 12-13y; and (2) how the domain-specific composition of PA at 10-11y is associated with HRQOL at 10-11y and 12-13y, independent of total PA.

## Materials and methods

### Setting and procedures

The data source for this study was the Birth (B) cohort of the Longitudinal Study of Australian Children (LSAC). LSAC is a national longitudinal research project managed by the Australian Department of Social Services which aims to “identify policy opportunities for improving support for children and their families, and identifying the opportunities for early intervention” [44]. Data are publicly available upon application [45]. LSAC data collection processes were approved by the ethics committee of the Australian Institute of Family Studies, and informed consent was provided by parents or legal guardians on behalf of participating children at baseline [46]. The use of data in the present study was approved by the University of Wollongong Human Research Ethics Committee (2022/256).

### Participants

A nationally-representative sample of participants was recruited from the Australian Medicare database at the beginning of the LSAC study (2004) and have been followed-up every two years to date. Participants were recruited via a two-stage clustered design, involving the random selection of postcodes then families [44]. Stratified selection was used to ensure proportional inclusion of participants of each Australian state and territory across major city and regional/remote areas. Eligible children were born between March 2003 and February 2004 [44]. A total of 5107 families were recruited at baseline (57% of those invited to participate). LSAC utilize a variety of methods to follow-up participants between waves including mail, telephone and in-person visits [44]. The present study uses LSAC B cohort data collected during Wave 6 (2014) and Wave 7 (2016).

### Measurement of HRQOL

The main outcome variables in this study were HRQOL domains, measured at 10-11y and 12-13y using the Pediatric Quality of Life Inventory (PedsQL™) [47]. PedsQL™ consists of 23 items that assess four generic core scales: physical functioning (8 items; e.g., “How often would you say this child has had a problem with walking?”), emotional functioning (5 items; e.g., “How often would you say this child has had a problem with feeling afraid or scared?”), social functioning (5 items; e.g., “How often would you say this child has had a problem with getting along with other children?”) and school functioning (5 items; e.g., “How often would you say this child has had a problem with paying attention in class?”) [47]. Each item is measured on a 5-point scale ranging from 0 (never a problem) to 4 (almost always a problem). To account for missing data, the generic core scale scores are derived by summing the items and then dividing by the number of items in the scale, as per published instructions. Scales are then linearly transformed and reverse-scored from 0–100, with higher scores corresponding with better functioning [47]. PedsQL™ also provides a psychosocial functioning summary scale (calculated as the mean of the items in the emotional, social and school subscales) and a total functioning summary scale (calculated as the mean of all items) [47]. The key outcomes in the present study were the physical functioning generic core scale, the psychosocial functioning summary scale and the total functioning summary scale. Findings for the components of psychosocial functioning (emotional, social and school) are presented in Additional file 1 and Additional file 2. The parent-reported version of PedsQL™ was used in LSAC, and this has been shown to have strong internal consistency reliability for physical functioning (α = 0.88), psychosocial functioning (α = 0.86) and total functioning (α = 0.90) [47]. These scales have also been validated against indicators such as the extent to which the child’s HRQOL interfered with the parent’s work routine, with moderate correlations found for physical functioning (*r* = –0.31), psychosocial functioning (*r* = –0.43) and total functioning (*r* = –0.44). To allow greater interpretability, PedsQL™ scores have been converted to percentages in the present study by dividing them by the maximum possible score of 100. Varni and colleagues previously reported minimum clinically important differences for the PedsQL™ scales which represent the smallest difference in scores that patients are likely to perceive as beneficial. As percentages, these thresholds are + 6.9% for physical HRQOL, + 5.5% for psychosocial HRQOL and + 4.5% for total HRQOL [48].

### Measurement of domain-specific PA

It can be challenging to accurately capture habitual time spent in PA domains, as device-based methods such as accelerometers cannot differentiate between these domains. Subjective methods of capturing activity need to be used [49]. Surveys and questionnaires on average times (e.g., over the last week or month) spent in behaviours of interest are often used for PA domains; however, these have the limitation of not being bounded by the 24-h day, i.e., the total time spent across PA domains could potentially sum to more than 24 h. Time-use recalls or time-use diaries attempt to overcome this limitation by capturing activities within the context of a complete 24-h day [49]. However, 24-h instruments are more burdensome to participants, and often require trained interviewers to assist the recall process. Thus, the feasibility of capturing multiple days is limited, and activity data are specific to the day(s) that was recalled.

The main predictor variables in this study were the durations of domain-specific PA participation (min/day), measured using a one-day time-use diary (TUD). Participants received a structured paper diary with instructions to record activities in their own words for the day before their interview at 10-11y (between wake and sleep times) [50]. Participants who attended school on this day were told to record activities that occurred during school breaks but not during lessons (such as physical education (PE) lessons). Diary entries were then coded by interviewers using a predetermined coding framework during the home visit [50]. Interviewers were trained to prompt the child for additional information during this process, such as to fill gaps in the diary [50].

Variables corresponding with the duration of each PA domain were calculated based on specific TUD activity codes using a series of steps, consistent with our previous research [11]. Firstly, the duration of each activity was calculated as the difference between the start and end time. It was assumed that each activity ended at the start time of the next activity in sequence for each child, with the final activity ending at the child’s bed time. The total duration of each activity code was then aggregated for each participant. Although participants could record up to six activities concurrently, some domains (such as organized PA) seemed incongruent as a concurrent activity. For consistency, participation in domains of PA was based on the primary activity selected. An additional quality improvement process was also conducted prior to the present study which involved comparing TUD codes with free-text descriptions of activities included in the LSAC datasets. More details about this process have been provided in a previous study [51]. Additional file 3 defines the domains of PA that were used in the present study. It is important to note that only one day of activities was recorded by each participant. This may impact the validity of the measurement of time spent in PA domains, particularly if a child regularly participated in sport or active chores on a different day of the week than was assessed by the TUD.

### Measurement of other variables

Models controlled for potential confounding variables including sex, age in months, body mass index (BMI), pubertal status and socioeconomic position, as has been done previously [52]. Analyses also adjusted for TUD contextual variables (season and school attendance) that may have been related to participants’ PA time. BMI was calculated by LSAC and provided as a z-score based on the CDC growth charts [53] using the child’s height and weight as measured by LSAC interviewers. Pubertal status was assessed using the Petersen pubertal development scale, which asked the parent to assess the extent of the child’s development of body hair, growth spurt, skin changes, facial hair (males), voice changes (males), breast changes (females) and menarche (females) [54]. This scale has adequate reliability (α = 0.77) and has been validated against physicians’ assessments [54]. Socioeconomic position was determined using an LSAC-derived scale based on household income, occupational status of parents and educational attainment of parents [55]. This scale has been validated against indicators such as experiencing economic hardship and being a recipient of income support payments [56]. In terms of TUD contextual variables, season was derived from the month of interview and school attendance was included as a variable in LSAC datasets. Missing data for school attendance was imputed using the ‘school lessons’ TUD code (it was assumed that children attended school if this code was used).

### Analyses

Robust linear regressions were used to assess the relationship between HRQOL outcomes and: (1) the absolute duration of PA domains; and (2) the domain-specific PA composition. Initial data processing was performed using IBM SPSS Statistics (version 26; IBM Corp., Armonk, NY, USA). Data analyses were performed in R (version 1.4.1103; R Foundation for Statistical Computing, Vienna, Austria) using packages including ‘robCompositions’ and ‘robustbase’. Robust models were used due to heteroscedasticity in residuals and potentially outlying observations. Due to the likely rounding of TUD entries to the nearest 5 min, all values for PA domains were consistently rounded to 5-min increments. In addition, TUDs were included in analyses if they had more than 12 h of activities between wake and bedtimes, similar to the approach of Chong and colleagues [56]. TUDs below this threshold were likely to be incomplete or to have unusual sleep patterns (e.g. due to sickness). Effect sizes and confidence intervals were used to report and interpret the results, with the exception of the ANOVA table of model fit for the overall compositional models as described further below. P-values for the full models are provided in Additional file 1 and Additional file 2.

In the first set of analyses, the absolute durations of each PA domain (min/day) were individually included in regression models to predict HRQOL scores at 10-11y (cross-sectional) and at 12-13y (longitudinal), while controlling for potential confounding variables. Longitudinal models additionally controlled for HRQOL scores at 10-11y to determine the additional future benefit associated with participation in PA domains.

The second set of analyses tested associations between the domain-specific PA composition at 10-11y and HRQOL scores at 10-11y (cross-sectional) and at 12-13y (longitudinal). Prior to conducting regressions, a compositional data analysis approach (CoDA) was used to generate four sets of isometric log-ratio (*ilr*) coordinates, each representing time spent in one PA domain at 10-11y relative to all remaining domains [40]. To avoid undefined values when calculating ilrs, zero values in PA domains were classified as “rounded” under the assumption that every child would eventually accumulate time in each PA domain if the recording period was longer. Zeros were replaced using a multiplicative replacement strategy (65% of the smallest recorded duration, i.e., 1 min) [57]. The set of *ilrs* were then included in robust linear regression models to predict HRQOL scores, while controlling for potential confounding variables. The multiplicative total of PA domains was included in models to control for the total amount of PA (min/day) at 10-11y, as per the ‘CoDA with a total’ approach (tCoDA) [42]. Longitudinal compositional models also controlled for the HRQOL scores at 10-11y as was done in non-compositional models. The ANOVA table of model fit was used to determine whether the set of *ilrs* collectively had a statistically significant association with HRQOL outcomes. The robust regression coefficients were used to perform compositional reallocations of 30 min to one activity domain (which we considered a feasible behaviour change), taking time proportionately (pro rata) from the remaining activity domains [40]. Additionally, HRQOL outcomes associated with incrementally increasing the share of PA time spent in each domain in 5% units were plotted to aid meaningful interpretation of effect sizes. These visualisations were based on predictive formulas that estimated outcomes for every possible theoretical iteration of domain-specific PA in 5% increments within the bounds of the study sample, similar to the approach of Dumuid and colleagues [58].

## Results

**Fig. 1:**
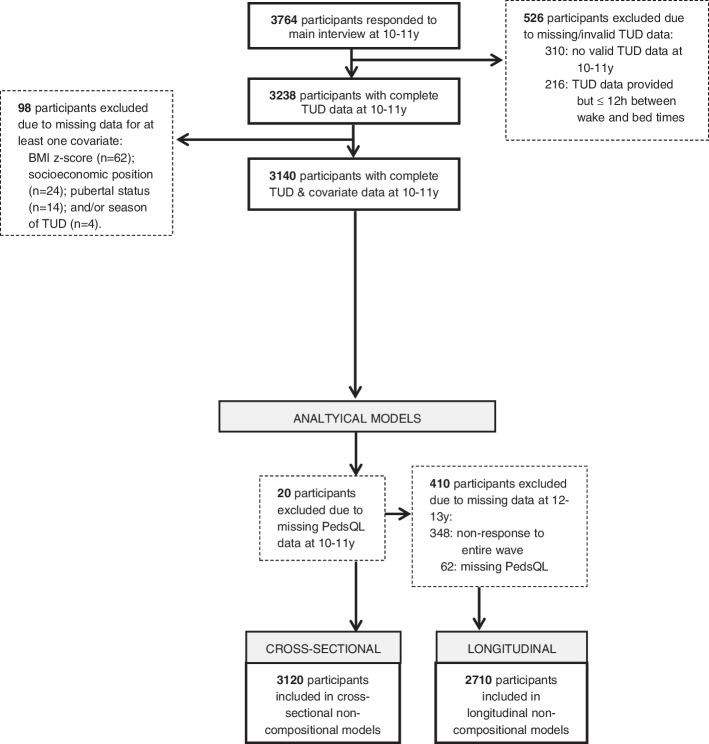
Flowchart showing the reasons for exclusion from the analytic samples

A total of 3764 participants took part in the main LSAC interview at 10-11y. Of these, 3120 participants (83%) were included in at least one analytic sample in the present study (see Fig. 1). Participants were excluded if they had missing data or if their time-use diary data were invalid. Compared with participants who were included in at least one analytic sample, those who were excluded from all final models were slightly older, of lower socioeconomic position and were less likely to complete the TUD on a school day (see Table 1). The average follow-up duration for participants included in at least one longitudinal model was 24.6 months (SD = 3.1 months, *n* = 2710).

**Table 1:** Characteristics of participants included in at least one analytic sample and the total sample

	**Included in ≥ 1 analytic sample (*n* = 3120)**	**All participants at 10-11y (*n* = 3764) **^**a**^
Males, n (%)	1592 (51.0%)	1929 (51.2%)
Age in months, mean (SD)	130.5 (4.0) ^b^	130.6 (4.0)
Socioeconomic position, z-scored, mean (SD)	0.0 (1.0) ^b^	0.0 (1.0)
BMI, z-score	0.3 (1.2)	0.3 (1.2)
Pubertal development score ^c^	1.7 (0.5)	1.7 (0.5)
Attended school on day of TUD, n (%)	1602 (51.3%) ^b^	1683 (48.7%)
Season of measurement, n (%)		
Summer	61 (2.0%)	72 (2.1%)
Autumn	774 (24.8%)	848 (24.5%)
Winter	1227 (39.3%)	1380 (39.8%)
Spring	1058 (33.9%)	1166 (33.6%)
PA domain participation at 10-11y (min/day)		
Non-organized PA, mean (SD)	67.2 (81.4)	66.9 (81.4)
Organized PA, mean (SD)	27.5 (55.5)	27.2 (55.0)
Active transport, mean (SD)	9.7 (27.2)	9.9 (27.8)
Active chores/work, mean (SD)	15.4 (38.7)	15.6 (39.4)
HRQOL outcomes at 10-11y (PedsQL™ scores)		
Physical functioning, mean (SD)	84.3 (14.8)	83.9 (15.4)
Psychosocial functioning, mean (SD)	77.4 (14.9)	76.9 (15.4)
Total functioning, mean (SD)	80.5 (13.1)	80.0 (13.6)
HRQOL outcomes at 12-13y (PedsQL™ scores)		
Physical functioning, mean (SD)	84.1 (15.2)	83.9 (15.5)
Psychosocial functioning, mean (SD)	78.9 (14.7)	78.8 (14.8)
Total functioning, mean (SD)	81.2 (13.2)	81.1 (13.4)

*HRQOL* health-related quality of life, *n* number of participants, *PA* physical activity, *PedsQL™* Pediatric Quality of Life Inventory, *%* proportion of sample, *SD* standard deviation, *TUD* time use diary. Percentages may add to more than 100% due to rounding

^a^ Variable-specific missing data for all participants at 10-11y: socioeconomic position (*n* = 57), BMI z-score (*n* = 192), pubertal development score (*n* = 57), school attendance on day of TUD (*n* = 309), season of measurement (*n* = 298), PA domain participation (*n* = 525; one additional value was missing for active transport due to an implausibly large value that was removed), PedsQL™ scores at 10-11y (*n* = 111). PedsQL™ scores at 12-13y (*n* = 965)

^b^ Participants with available covariate data who were excluded from all models were slightly older (131.1 months, *p* = 0.001, *n* = 644), of lower socioeconomic position (-0.2, *p* < 0.001, *n* = 587) and were less likely to have completed the TUD on a school day (24.2%, *p* < 0.001, *n* = 335), compared with those included in at least one model (*n* = 3120)

^c^ The pubertal development scale ranged from 1 (least developed) to 4 (most developed)

### Non-compositional models

**Fig. 2:**
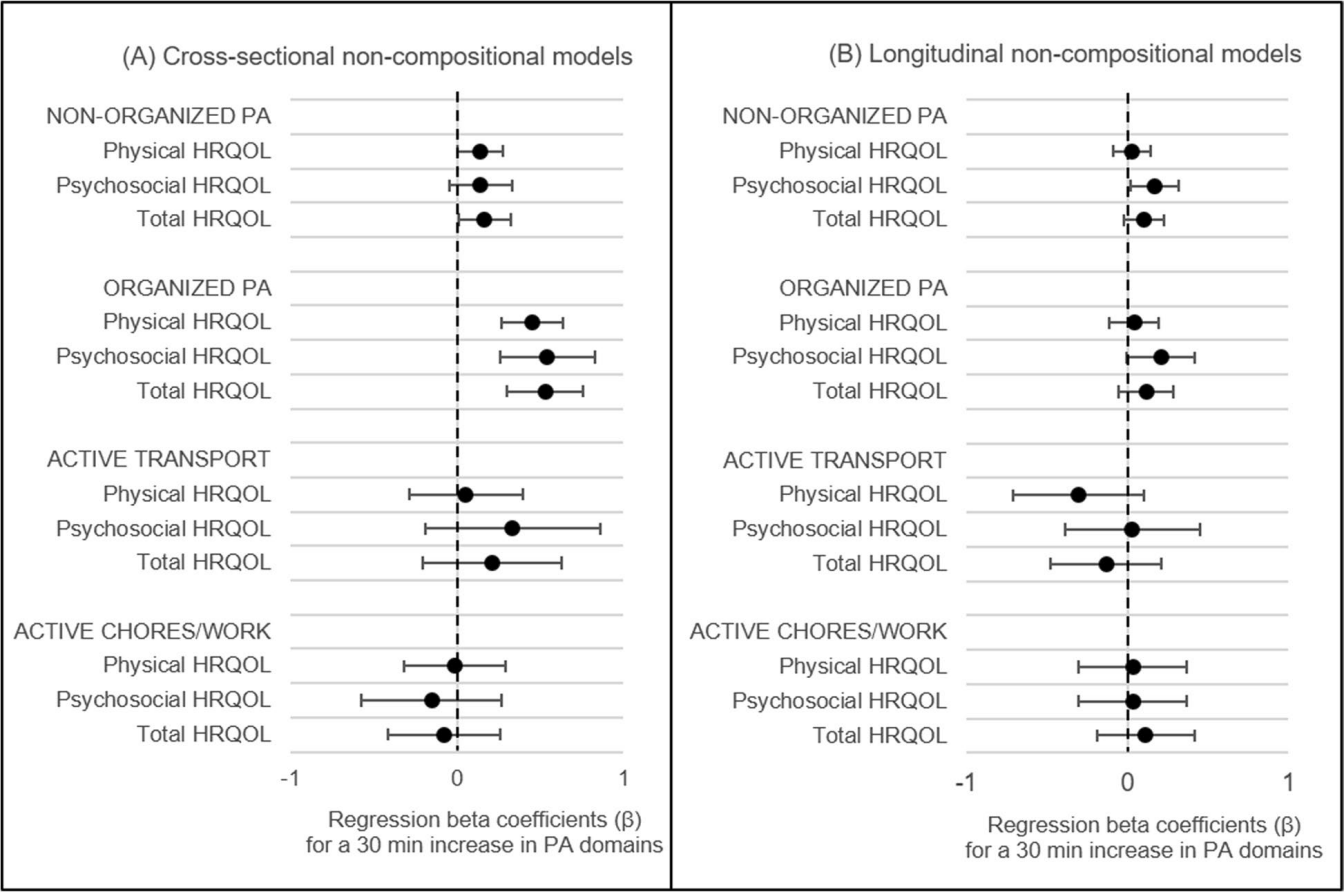
Associations between PA domain duration and HRQOL outcomes. Associations between a + 30 min difference in duration of domain-specific PA participation at 10-11y and HRQOL outcomes at: 10-11y (**A**); and 12-13y (**B**), showing coefficients and 95% confidence intervals. All models controlled for sex, age, BMI, pubertal status, socioeconomic position and TUD contextual variables (season and school attendance) at 10-11y. Compositional models additionally controlled for the multiplicative total of PA. Longitudinal models additionally controlled for HRQOL scores at 10-11y.For compositional models, the + 30 min in a PA domain is relative to pro-rata reductions in the remaining PA domains

A total of 3120 participants were included in models that tested cross-sectional associations between the absolute duration of PA domains and HRQOL outcomes at 10-11y. The results of the models are detailed in Table 2 and visualised in Fig. 2. Results showed that the durations of organized PA, and to a lesser extent, non-organized PA were positively associated with HRQOL at 10-11y. A 30-min increase in organized PA per day was associated with slightly better physical HRQOL (β =  + 0.45%, 95%CI =  + 0.27%, + 0.63%), psychosocial HRQOL (β =  + 0.55%, 95%CI =  + 0.27%, + 0.83%) and total HRQOL at 10-11y (β =  + 0.54%, 95%CI =  + 0.31%, + 0.76%). In addition, a 30-min increase in non-organized PA per day was associated with marginally better physical HRQOL (β =  + 0.14%, 95%CI =  + 0.0004%, + 0.28%) and total HRQOL at 10-11y (β =  + 0.17%, 95%CI =  + 0.01%, + 0.32%). The duration of other PA domains were not associated with HRQOL outcomes at 10-11y.

**Table 2:** Associations between domain-specific PA duration at 10-11y and HRQOL outcomes at 10-11y and 12-13y

PA domain	Cross-sectional models (*n* = 3120)^a^		Longitudinal models (*n* = 2710)^a^	
	** + 30 min**		** + 30 min**	
	**Estimate**	**95% CI**	**Estimate**	**95% CI**
**Physical functioning**^**b**^				
Non-organized PA	** + 0.14%**	** + 0.001%, + 0.28%**	+ 0.01%	–0.10%, + 0.13%
Organized PA	** + 0.45%**	** + 0.27%, + 0.63%**	+ 0.04%	–0.11%, + 0.20%
Active transport	+ 0.05%	–0.29%, + 0.39%	–0.29%	–0.70%, + 0.11%
Active chores/work	–0.01%	–0.31%, + 0.29%	+ 0.02%	–0.31%, + 0.36%
**Psychosocial functioning**^**c**^				
Non-organized PA	+ 0.14%	–0.05%, + 0.33%	** + 0.17%**	** + 0.03%, + 0.32%**
Organized PA	** + 0.55%**	** + 0.27%, + 0.83%**	+ 0.21%	–0.003%, + 0.41%
Active transport	+ 0.33%	–0.20%, + 0.86%	+ 0.05%	–0.37%, + 0.47%
Active chores/work	–0.15%	–0.57%, + 0.27%	+ 0.05%	–0.28%, + 0.39%
**Total functioning**^**d**^				
Non-organized PA	** + 0.17%**	** + 0.01%, + 0.32%**	+ 0.10%	–0.02%, + 0.22%
Organized PA	** + 0.54%**	** + 0.31%, + 0.76%**	+ 0.12%	–0.05%, + 0.28%
Active transport	+ 0.21%	–0.21%, + 0.63%	–0.12%	–0.46%, + 0.23%
Active chores/work	-0.07%	–0.41%, + 0.26%	+ 0.12%	–0.18%, + 0.42%

*PA* physical activity, *HRQOL* health related quality of life, measured using PedsQL™ scales, *n* number of participants, *β* model coefficient, *CI* confidence interval. Differences significant at *p* < 0.05 are shown in bold

^a^Models adjusted for sex, age, BMI, pubertal status, socioeconomic position and TUD contextual variables (season and school attendance) at 10-11y. Longitudinal models additionally controlled for HRQOL scores at 10-11y. Unadjusted estimates are provided in Additional file 1

^b^ + 6.9% is considered a clinically important difference for physical HRQOL

^c^ + 5.5% is considered a clinically important difference for psychosocial HRQOL

^d^ + 4.5% is considered a clinically important difference for total HRQOL

Longitudinal models (*n* = 2710) revealed that a 30-min increase in non-organized PA per day at 10-11y was associated with a 0.17% (95%CI =  + 0.03%, + 0.32%) increase in psychosocial HRQOL at 12-13y. The longitudinal association between the duration of organized PA at 10-11y and psychosocial HRQOL at 12-13y was of a similar magnitude, although the 95% confidence interval slightly intersected zero (β =  + 0.21%, 95%CI = –0.003%, + 0.41%). All associations observed in both cross-sectional and longitudinal models fell below the minimum clinically important differences for the PedsQL™ scales as proposed by Varni and colleagues [48].

### Compositional models

**Fig. 3:**
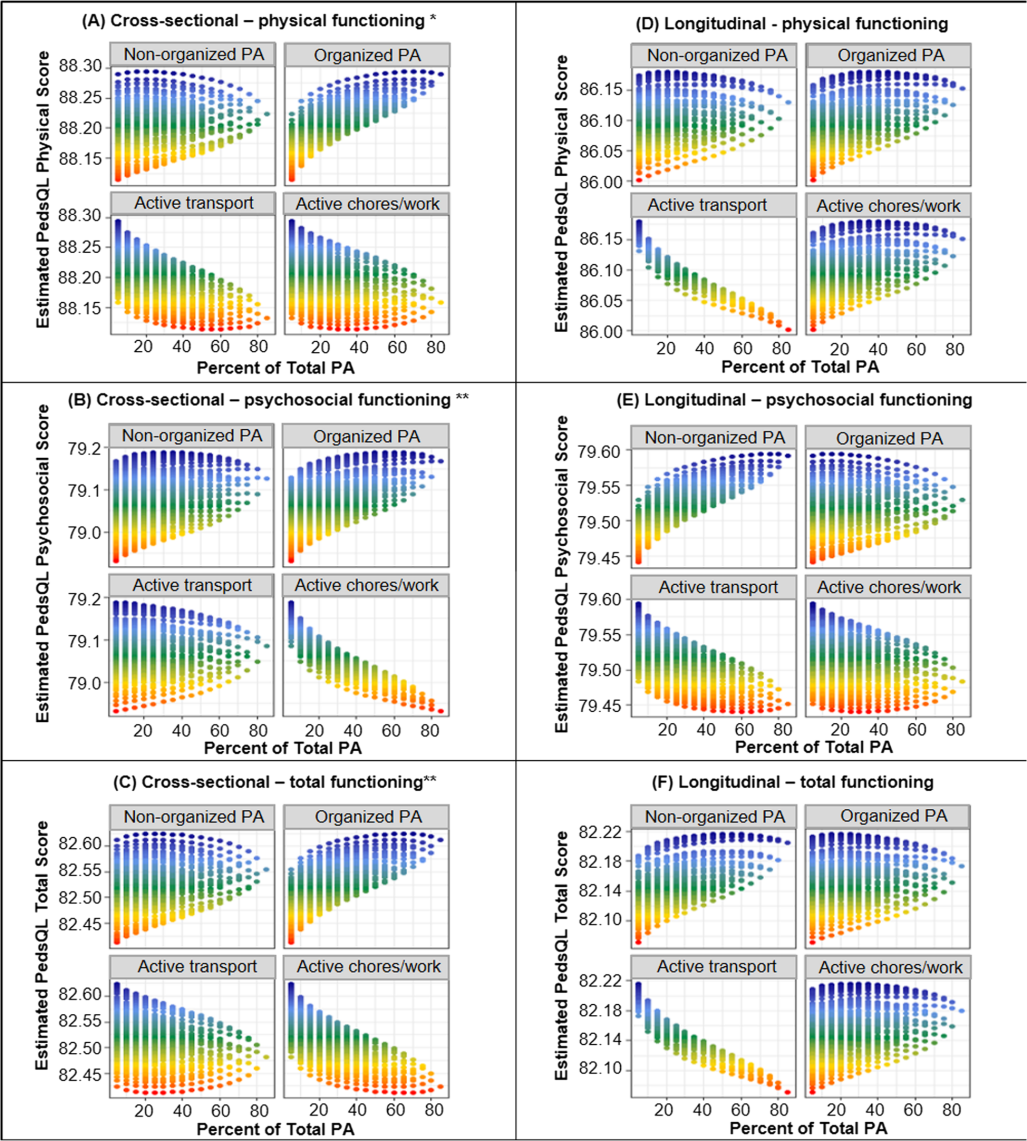
Incremental changes in PA composition spent in each domain of PA and predicted HRQOL outcomes. Relationship between incrementally increasing the share of PA time spent in each domain of PA at 10-11y and predicted HRQOL outcomes at: 10-11y (A–C); and 12-13y (D–F). Each data point represents the predicted HRQOL outcomes for a given permutation of activity compositions (in 5% increments). Predictions controlled for sex, age, BMI, pubertal status, socioeconomic position, TUD contextual variables (season and school attendance) and the multiplicative total of PA at 10-11y. Longitudinal models additionally controlled for HRQOL scores at 10-11y. Please carefully note the differing vertical axes between individual charts in this figure (average physical HRQOL scores were several points higher than psychosocial HRQOL scores in this sample which prevented the depiction of discernible trends on a common vertical axis). Asterisks after individual chart headings indicate that the set of *ilrs* collectively had a statistically significant association with HRQOL outcomes, based on the ANOVA table of model fit (* = *p* < 0.05)

A total of 3120 participants were included in models that tested cross-sectional associations between the composition of PA and HRQOL outcomes at 10-11y. The results of the models are detailed in Table 3 and visualised in Fig. 3. The overall composition of PA was related to physical HRQOL (χ^2^ = 9.01, *p* = 0.03), psychosocial HRQOL (χ^2^ = 11.37, *p* = 0.01) and total HRQOL at 10-11y (χ^2^ = 11.29, *p* = 0.01), independent of total PA durations. Differences were particularly noted regarding the share of PA time spent in organized PA and active chores/work. Relative to time spent in other PA domains, increased time (+ 30 min) spent in organized PA was positively associated with physical HRQOL (+ 0.32%, 95%CI =  + 0.01%, + 0.63%), psychosocial HRQOL (+ 0.41%, 95%CI =  + 0.11%, + 0.72%) and total HRQOL at 10-11y (+ 0.39%, 95%CI =  + 0.12%, + 0.66%). Conversely, increased time spent in active chores/work relative to other PA domains was negatively associated with psychosocial HRQOL (–0.52%, 95%CI = –0.91%, –0.14%). Similar to the results of non-compositional models, all the associations observed in the compositional models fell below the minimum clinically important differences for the PedsQL™ scales as proposed by Varni and colleagues [48].

**Table 3:** Compositional associations between PA composition at 10-11y and HRQOL outcomes at 10-11y and 12-13y

PA domain	Cross-sectional models (*n* = 3120)^a^		Longitudinal models (*n* = 2710)^a^	
	** + 30 min, relative to remaining activities**		** + 30 min, relative to remaining activities**	
	**Estimate**	**95% CI**	**Estimate**	**95% CI**
**Physical functioning**				
Non-organized PA	+ 0.10	-0.36, + 0.56	-0.02	-0.46, + 0.41
Organized PA	** + 0.32**	** + 0.01, + 0.63**	+ 0.18	-0.11, + 0.47
Active transport	-0.34	-0.82, + 0.13	-0.29	-0.72, + 0.14
Active chores/work	-0.11	-0.5, + 0.27	+ 0.09	-0.27, + 0.45
**Psychosocial functioning**				
Non-organized PA	+ 0.10	-0.36, + 0.55	+ 0.18	-0.20, + 0.55
Organized PA	** + 0.41**	** + 0.11, + 0.72**	+ 0.06	-0.19, + 0.31
Active transport	-0.01	-0.47, + 0.46	-0.15	-0.52, + 0.23
Active chores/work	**-0.52**	**-0.91, -0.14**	-0.06	-0.37, + 0.26
**Total functioning**				
Non-organized PA	+ 0.10	-0.30, + 0.50	+ 0.07	-0.27, + 0.41
Organized PA	** + 0.39**	** + 0.12, + 0.66**	+ 0.06	-0.16, + 0.29
Active transport	-0.19	-0.60, + 0.22	-0.23	-0.56, + 0.11
Active chores/work	-0.33	-0.67, + 0.01	+ 0.09	-0.19, + 0.37

*PA* physical activity, *HRQOL* health related quality of life, measured using PedsQL™ scales, *n* number of participants, *CI* confidence interval. Differences significant at *p* < 0.05 are shown in bold

^a^ Models adjusted for sex, age, BMI, pubertal status, socioeconomic position, TUD contextual variables (season and school attendance) and the multiplicative total of PA at 10-11y. Longitudinal models additionally controlled for HRQOL scores at 10-11y. Unadjusted estimates are provided in Additional file 2

Longitudinal compositional models (*n* = 2710) revealed that there were no statistically significant associations between the composition of PA domains at 10-11y and physical HRQOL (χ^2^ = 5.43, *p* = 0.14), psychosocial HRQOL (χ^2^ = 1.38, *p* = 0.73) and total HRQOL at 12-13y (χ^2^ = 2.35, *p* = 0.50).

Additional files 1 and 2 provide the full results of all models, including unadjusted estimates and results for covariates.

## Discussion

This study used non-compositional and compositional models to explore associations between PA domain participation and HRQOL outcomes during childhood and adolescence. Despite differences in how the data were handled, both analytical approaches revealed consistent patterns of weak beneficial cross-sectional associations for organised PA (which were attenuated in longitudinal models). Specifically, non-compositional models indicated that the absolute duration of organized PA at 10-11y was consistently, albeit weakly, associated with HRQOL outcomes at 10-11y but did not necessarily predict HRQOL at 12-13y. The absolute duration of non-organized PA at 10-11y had a weak positive association with physical and total HRQOL at 10-11y and also predicted marginally better psychosocial HRQOL at 12-13y. Cross-sectional compositional models indicated that, relative to time spent in other PA domains, organized PA had weak positive associations with all HRQOL domains and active chores/work had weak negative associations with psychosocial and total functioning at 10-11y. However, the overall composition of PA at 10-11y did not longitudinally predict HRQOL outcomes at 12-13y.

It should be noted that effect sizes in this study tended to be small. The largest effect size was in the non-compositional association between organized PA and psychosocial HRQOL at 10-11y (β_+30 min/day_ =  + 0.55%), which was weaker than the associations for some covariates in this model such as pubertal development (β = –1.99%), socioeconomic position (β =  + 0.73%) and sex (female: β = –0.62%) (see Additional files 1 and 2). These findings appear to be congruent with a previous meta-analysis by Marker and colleagues [30] that reported negligible associations between overall PA and parent-reported HRQOL based on descriptive studies of children and adolescents (Hedges’ g = 0.115). There are also some similarities between our findings and those of previous studies that tested associations between the duration of domain-specific PA and HRQOL. For example, a previous Australian longitudinal study by Vella and colleagues [37] reported weak associations between organized sport at 10y and physical HRQOL (β_+30 min/day_ =  + 0.60%) and social HRQOL at 12y (β_+30 min/day_ =  + 0.90%), although baseline HRQOL was not controlled for in this study. Weak associations have also been reported between sport participation (hours/week) and physical HRQOL (*r* = 0.15) and social HRQOL (*r* = 0.17) among Iranian girls but not boys aged 14-17y [36]. Finally, a study of Australian youth aged 10-13y by Tsiros and colleagues [19] reported positive associations between organized sport (min/day) and HRQOL (VIP > 1), while there was a marginal positive association between active play (min/day) and HRQOL among boys (just below VIP = 1). Finally, it should be noted that all effect sizes in the present study fell below Varni and colleagues’ criteria for minimum clinically important differences for the PedsQL™ scales (+ 6.9% for physical HRQOL, + 5.5% for psychosocial HRQOL and + 4.5% for total HRQOL) [48]. This means that although some domains of PA may have marginal associations with HRQOL, any improvements in the lived experiences of youth are likely to be below a perceptible level [48].

Time spent in organized PA was consistently related to HRQOL at 10-11y in the present study, although effect sizes were weak. Positive associations between organized PA and HRQOL are plausible, as previous reviews have reported beneficial associations between organized PA and outcomes such as cardiovascular fitness [13], mental health [12], peer relations [59] and self-esteem [60]. Similar trends were not observed in longitudinal models in the present study, apart from a possible marginal association between the absolute duration of organized PA at 10-11y and psychosocial HRQOL at 12-13y. This suggests that although organized PA could potentially predict some aspects of future HRQOL, it is possible that other mechanisms may have contributed at least in part to the cross-sectional associations in our study. For example, organized PA has been shown to have bidirectional associations with some physical [61] and mental health indicators [62], so it is possible that the cross-sectional associations occurred at least in part because youth who participated in more organized PA already had better HRQOL. Compensation with other movement behaviours may also be an explanation; for example, organized sport may displace sedentary behavior, which could, in turn, lead to an overall improvement in HRQOL [63]. However, it is also possible that organized PA might have some contemporaneous benefits even though these may not necessarily translate to predicting future HRQOL.

The duration of non-organized PA also had weak positive associations with some outcomes in the present study. In cross-sectional models, the duration of non-organized PA was positively associated with physical and total HRQOL. These positive associations are plausible based on previous reviews that reported positive associations between non-organized PA and physical outcomes such as motor skills [15, 64] and cardiovascular fitness [64]. The duration of non-organized PA also predicted marginally better psychosocial health in longitudinal models. It is possible that some types of non-organized PA may enable youth to form connections with others, similar to the function of active play during early childhood [65]. In addition, the self-directed and freely-chosen nature of non-organized PA may allow youth to have greater flexibility to choose enjoyable types of PA that suit their interests [6]. This may have a beneficial effect, as engaging in enjoyable activities such as hobbies have been linked to decreased depressive symptoms among adults [66]. However, as discussed previously, the small effect sizes suggest that benefits are likely to be below a perceptible level in the lived experiences of youth [48].

This study also identified some associations between the overall composition of PA and HRQOL outcomes. Cross-sectional compositional models revealed that organized PA was positively associated with all domains of HRQOL and active chores/work was negatively associated with psychosocial and total functioning, relative to other PA domains. In the case of active chores/work, it should be noted that the negative association does not necessarily mean that time spent in this domain is ‘bad’ for HRQOL. Rather, the association could mean that this domain is ‘less beneficial’ for HRQOL relative to other domains of PA. It is possible that youth who spent a greater share of PA time participating in active chores/work may have had additional responsibilities such as caring for siblings or working in a part-time job [67, 68]. These additional responsibilities may have ‘crowded out’ the available leisure time for other beneficial pursuits and resulted in greater time pressures [67, 68]. However, it should be noted that active chores/work did not predict HRQOL in longitudinal models. In fact, none of the longitudinal compositional models were statistically significant which suggests that the overall composition of PA was not particularly important for predicting future HRQOL outcomes in the present sample.

Based on the results of this study, a case might be made for the development of future health promotion strategies to encourage participation in organized and non-organized PA among youth. However, due to the small effect sizes in our study, these strategies should only form part of a broader suite of approaches designed to improve HRQOL. Practitioners could potentially encourage participation in these domains of PA while also targeting other behaviours that may be linked with HRQOL such as improved sleep [69] or reduced internet/smartphone use [70].

To the authors’ knowledge, this was the first known study to concurrently test associations between participation in the domains of organized PA, non-organized PA, active transport and active chores/work with a health outcome among children, and the first to use both compositional and non-compositional approaches to explore this topic. The consistency of patterns observed across both analytical methodologies supports the robustness of the findings. The compositional approach allows all PA domains to be included in a single model, and considers an increase in one domain relative to decreases in other domains, which may be more relevant to real life scenarios where time for PA is limited. However, the non-compositional approach is computationally simpler, and the interpretation of model parameters is more direct and straightforward than for compositional models. We simulated what we considered a feasible behavior change (i.e., an increase in duration of 30 min), however it should be noted that some activity behaviors may be more difficult to change than others. Within the current sample, a 30-min change represented different fractions of the observed standard deviations (SD): 37% for non-organized PA, 55% for organized PA, 107% for active transport and 75% for active chores/work.

To our knowledge this was also the first known study to test longitudinal HRQOL associations for certain PA domains, such as non-organized PA and active chores/work. However, this study also has some limitations. PA was self-reported via youth TUDs and this may have led to recall bias and overestimation of PA participation [71]. However, self-report methods were necessary because more objective methods such as accelerometry are not able to easily capture information about PA context [72]. Additionally, the LSAC study only used a one-day TUD and this may not have captured variation in participants’ routines between days of the week. This may have particularly affected the estimate of organized PA, which in this study captured extra-curricular activities such as sport. Such activities may have been missed for a participant if the organized PA was not completed on the day of the TUD. Despite this, analyses controlled for season and school attendance on the day of TUD completion, and 24-h TUDs have demonstrated promising validity [73] and reliability [74]. The LSAC TUD also did not include time in Physical Education (PE) classes, which may under-estimate time spent in organized physical activity, and potentially could impact the observed associations. However, as PE is a compulsory subject among Australian students at this age, we expect any associated error to be systematic rather than biased. Residual confounding from unmeasured or omitted variables may affect our findings (e.g., physical limitations for physical activity, environmental pollution, or neighborhood safety). Further, participants were instructed not to record activities during school lessons in the TUD (such as PE lessons). This means that LSAC TUD data may under-estimate the time spent in organized PA compared with other PA domains, which could potentially impact the associations observed in this study. It should also be noted that this was an observational study. Despite the inclusion of longitudinal analyses, an experimental intervention design would be required to test causal associations between variables. Finally, LSAC is a ‘closed’ longitudinal study which means that no new participants have been recruited since baseline. Therefore the LSAC sample is likely to underrepresent some population groups, particularly immigrants arriving in Australia after 2004. These factors should be considered when applying the study results to populations in Australia or elsewhere.

## Conclusions

Non-compositional models revealed that the absolute duration of organized PA was weakly associated with HRQOL outcomes at 10-11y but did not necessarily predict HRQOL at 12-13y. The absolute duration of non-organized PA had a weak positive association with physical and total HRQOL at 10-11y and also predicted marginally better psychosocial HRQOL at 12-13y. Cross-sectional compositional models indicated that, relative to time spent in other PA domains, organized PA had weak positive associations with all HRQOL domains and active chores/work had weak negative associations with psychosocial and total functioning at 10-11y. However, the overall composition of PA did not longitudinally predict HRQOL outcomes at 12-13y. The effect sizes of all associations were small, which suggests that domain-specific PA participation is unlikely to make a perceptible difference in the lived experiences of young people’s HRQOL. Although health promotion strategies could potentially seek to encourage participation in organized and non-organized PA, this should only form part of a broader suite of measures.

## Supplementary Information

**Additional file 1.** Full results of non-compositional models. Provides the full results of all non-compositional models, including subcomponents of psychosocial health-related quality of life, unadjusted estimates and covariates.

**Additional file 2.** Full results of compositional models. Provides the full results of all compositional models, including subcomponents of psychosocial health-related quality of life, unadjusted estimates and covariates.

**Additional file 3.** PA domain definitions. Provides definitions of each domain of physical activity used in this study.

## Data Availability

The datasets supporting the conclusions of this article are available by application via the DSS Longitudinal Studies Dataverse: http://dx.doi.org/10.26193/BAA3N6. Restrictions apply to the availability of these data, which were provided under license for the current study by the Australian Department of Social Services.

## Abbreviations

B Cohort: Birth cohort of LSAC, aged 0-1y at baseline
BMI: Body Mass Index
CoDA: Compositional Data Analysis
DSS: Australian Department of Social Services
HREC: Human Research Ethics Committee
HRQOL: Health-Related Quality of Life
ilr: Isotemporal Log-Ratio
LSAC: Longitudinal Study of Australian Children
LMVPA: Light, moderate and vigorous physical activity
MVPA: Moderate-to-vigorous physical activity
PA: Physical activity
PE: Physical education
PedsQL™: Pediatric Quality of Life Inventory
TUDs: Time-use diaries
VIP: Variable Importance in Projection
