# Supplementary material for: Cross-sectional and longitudinal associations of domain-specific physical activity composition with health-related quality of life in childhood and adolescence in Australia
Source: Int J Behav Nutr Phys Act. 2023 Jun 6;20:67. doi: 10.1186/s12966-023-01466-6 (PMC10242981; doi:10.1186/s12966-023-01466-6)
Supplement: Supplementary file 1 — Additional file 1. Full results of non-compositional models. Provides the full results of all non-compositional models, including subcomponents of psychosocial health-related quality of life, unadjusted estimates and covariates. [file 12966_2023_1466_MOESM1_ESM.docx]

**Cross-sectional and longitudinal associations of domain-specific physical activity composition with health-related quality of life in childhood and adolescence in Australia**

**Additional file 1:** Full results of non-compositional models

***Cross-sectional models***

**Table A1A: Associations between the absolute duration of non-organized PA at 10-11y and main HRQOL outcomes at 10-11y**

|  | **Physical HRQOL** | | | **Psychosocial HRQOL [a]** | | | **Total HRQOL [b]** | | |  |  |  |  |  |  |  |  |  |  |  |  |
| --- | --- | --- | --- | --- | --- | --- | --- | --- | --- | --- | --- | --- | --- | --- | --- | --- | --- | --- | --- | --- | --- |
|  | **β** | **95% CI** | ***p*** | **β** | **95% CI** | ***p*** | **β** | **95% CI** | ***p*** |  |  |  |  |  |  |  |  |  |  |  |  |
| **Unadjusted models** |  |  |  |  |  |  |  |  |  |  |  |  |  |  |  |  |  |  |  |  |  |
| Non–organized PA (+30 min/day) [c] | 0.14 | 0.00, 0.27 | 0.048 | 0.14 | –0.05, 0.32 | 0.153 | 0.18 | 0.02, 0.33 | 0.025 |  |  |  |  |  |  |  |  |  |  |  |  |
| **Adjusted models** |  |  |  |  |  |  |  |  |  |  |  |  |  |  |  |  |  |  |  |  |  |
| Non–organized PA (+30 min/day) [c] | 0.14 | 0.00, 0.28 | 0.049 | 0.14 | –0.05, 0.33 | 0.151 | 0.17 | 0.01, 0.32 | 0.037 |  |  |  |  |  |  |  |  |  |  |  |  |
| Age (months) | 0.03 | –0.07, 0.13 | 0.574 | 0.07 | –0.07, 0.20 | 0.131 | 0.04 | –0.07, 0.16 | 0.457 |  |  |  |  |  |  |  |  |  |  |  |  |
| Sex (female) | –0.25 | –1.14, 0.63 | 0.578 | –0.56 | –1.76, 0.64 | 0.363 | –0.53 | –1.55, 0.49 | 0.312 |  |  |  |  |  |  |  |  |  |  |  |  |
| BMI (z–score) | –0.77 | –1.12, –0.43 | <0.001 | –0.33 | –0.79, 0.13 | 0.155 | –0.69 | –1.11, –0.26 | 0.001 |  |  |  |  |  |  |  |  |  |  |  |  |
| Pubertal development [d] | –1.55 | –2.58, –0.53 | 0.003 | 2.05 | –3.40, –0.70 | 0.003 | –1.89 | –3.04, –0.74 | 0.001 |  |  |  |  |  |  |  |  |  |  |  |  |
| Socioeconomic position (z–score) | 0.32 | –0.06, 0.71 | 0.101 | 0.81 | 0.26, 1.36 | 0.004 | 0.79 | 0.34, 1.24 | 0.001 |  |  |  |  |  |  |  |  |  |  |  |  |
| School attendance on day of TUD (No) | –1.07 | –1.85, –0.29 | 0.007 | –1.52 | –2.59, –0.45 | 0.005 | –1.33 | –2.22, –0.44 | 0.003 |  |  |  |  |  |  |  |  |  |  |  |  |
| Season of measurement [e] | 0.24 | –0.10, 0.58 | 0.161 | 0.23 | –0.22, 0.68 | 0.308 | 0.21 | –0.16, 0.59 | 0.271 |  |  |  |  |  |  |  |  |  |  |  |  |
| PA = physical activity; HRQOL = health related quality of life, measured using PedsQL scales; β = model coefficient; CI = confidence interval. | | | | | | | | | | | | | | | | |  |  |  |  |  |
| a. The psychosocial HRQOL summary scale is a composite of social, emotional and school HRQOL | | | | | | | | | | | |  |  |  |  |  |  |  |  |  |  |
| b. The total HRQOL summary scale is a composite of physical, social, emotional and school HRQOL | | | | | | | | | | | |  |  |  |  |  |  |  |  |  |  |
| c. The coefficients presented in this table for this domain of PA represent the predicted effect associated with an increase of 30 min/day. | | | | | | | | | | | | | | | | | | | | | |
| d. The pubertal development scale ranged from 1 (least developed) to 4 (most developed). | | | | | | | | | | |  |  |  |  |  |  |  |  |  |  |  |
| e. Season of TUD completion was categorized as: 1= Winter; 2=Spring; 3=Summer; 4=Autumn. | | | | | | | | | | | |  |  |  |  |  |  |  |  |  |  |

**Table A1B: Associations between the absolute duration of non-organized PA at 10-11y and additional HRQOL outcomes at 10-11y**

|  | **Social HRQOL** | | | **Emotional HRQOL** | | | **School HRQOL** | | |  |  |  |  |  |  |  |  |  |  |  |  |
| --- | --- | --- | --- | --- | --- | --- | --- | --- | --- | --- | --- | --- | --- | --- | --- | --- | --- | --- | --- | --- | --- |
|  | **β** | **95% CI** | ***p*** | **β** | **95% CI** | ***p*** | **β** | **95% CI** | ***p*** |  |  |  |  |  |  |  |  |  |  |  |  |
| **Unadjusted models** |  |  |  |  |  |  |  |  |  |  |  |  |  |  |  |  |  |  |  |  |  |
| Non–organized PA (+30 min/day) [a] | 0.16 | –0.05, 0.37 | 0.128 | 0.11 | –0.11, 0.32 | 0.331 | 0.12 | –0.07, 0.31 | 0.231 |  |  |  |  |  |  |  |  |  |  |  |  |
| **Adjusted models** |  |  |  |  |  |  |  |  |  |  |  |  |  |  |  |  |  |  |  |  |  |
| Non–organized PA (+30 min/day) [a] | 0.16 | –0.05, 0.37 | 0.133 | 0.11 | –0.11, 0.34 | 0.323 | 0.18 | –0.01, 0.36 | 0.065 |  |  |  |  |  |  |  |  |  |  |  |  |
| Age (months) | 0.06 | –0.09, 0.21 | 0.449 | 0.05 | –0.10, 0.21 | 0.493 | –0.12 | –0.26, 0.01 | 0.072 |  |  |  |  |  |  |  |  |  |  |  |  |
| Sex (female) | –0.48 | –1.82, 0.86 | 0.484 | –0.42 | –1.78, 0.95 | 0.548 | 1.44 | 0.29, 2.60 | 0.014 |  |  |  |  |  |  |  |  |  |  |  |  |
| BMI (z–score) | –0.64 | –1.30, 0.03 | 0.061 | –0.07 | –0.54, 0.39 | 0.756 | –0.31 | –0.75, 0.12 | 0.156 |  |  |  |  |  |  |  |  |  |  |  |  |
| Pubertal development [b] | –2.45 | –3.97, –0.93 | 0.002 | –1.64 | –3.15, –0.13 | 0.034 | –1.49 | –2.77, –0.22 | 0.022 |  |  |  |  |  |  |  |  |  |  |  |  |
| Socioeconomic position (z–score) | 1.32 | 0.70, 1.95 | <0.001 | 0.22 | –0.40, 0.83 | 0.488 | 1.66 | 1.10, 2.21 | <0.001 |  |  |  |  |  |  |  |  |  |  |  |  |
| School attendance on day of TUD (No) | –1.41 | –2.62, –0.20 | 0.022 | –1.63 | –2.86, –0.40 | 0.009 | –1.27 | –2.30, –0.23 | 0.016 |  |  |  |  |  |  |  |  |  |  |  |  |
| Season of measurement [c] | 0.46 | –0.05, 0.97 | 0.078 | –0.04 | –0.56, 0.48 | 0.876 | 1.05 | 0.65, 1.46 | <0.001 |  |  |  |  |  |  |  |  |  |  |  |  |
| PA = physical activity; HRQOL = health related quality of life, measured using PedsQL scales; β = model coefficient; CI = confidence interval. | | | | | | | | | | | | | | | | |  |  |  |  |  |
| a. The coefficients presented in this table for this domain of PA represent the predicted effect associated with an increase of 30 min/day. | | | | | | | | | | | | | | | | | | | | | |
| b. The pubertal development scale ranged from 1 (least developed) to 4 (most developed). | | | | | | | | | | |  |  |  |  |  |  |  |  |  |  |  |
| c. Season of TUD completion was categorized as: 1= Winter; 2=Spring; 3=Summer; 4=Autumn. | | | | | | | | | | | |  |  |  |  |  |  |  |  |  |  |

**Table A2A: Associations between the absolute duration of organized PA at 10–11y and main HRQOL outcomes at 10–11y**

|  | **Physical HRQOL** | | | **Psychosocial HRQOL [a]** | | | **Total HRQOL [b]** | | |  |  |  |  |  |  |  |  |  |  |  |  |
| --- | --- | --- | --- | --- | --- | --- | --- | --- | --- | --- | --- | --- | --- | --- | --- | --- | --- | --- | --- | --- | --- |
|  | **β** | **95% CI** | ***p*** | **β** | **95% CI** | ***p*** | **β** | **95% CI** | ***p*** |  |  |  |  |  |  |  |  |  |  |  |  |
| **Unadjusted models** |  |  |  |  |  |  |  |  |  |  |  |  |  |  |  |  |  |  |  |  |  |
| Organized PA (+30 min/day) [c] | 0.55 | 0.37, 0.73 | <0.001 | 0.67 | 0.39, 0.96 | <0.001 | 0.67 | 0.44, 0.90 | <0.001 |  |  |  |  |  |  |  |  |  |  |  |  |
| **Adjusted models** |  |  |  |  |  |  |  |  |  |  |  |  |  |  |  |  |  |  |  |  |  |
| Organized PA (+30 min/day) [c] | 0.45 | 0.27, 0.63 | <0.001 | 0.55 | 0.27, 0.83 | <0.001 | 0.54 | 0.31, 0.76 | <0.001 |  |  |  |  |  |  |  |  |  |  |  |  |
| Age (months) | 0.04 | –0.06, 0.14 | 0.463 | 0.07 | –0.06, 0.21 | 0.292 | 0.05 | –0.06, 0.17 | 0.370 |  |  |  |  |  |  |  |  |  |  |  |  |
| Sex (female) | –0.32 | –1.20, 0.56 | 0.479 | –0.62 | –1.82, 0.58 | 0.314 | –0.60 | –1.61, 0.42 | 0.250 |  |  |  |  |  |  |  |  |  |  |  |  |
| BMI (z–score) | –0.74 | –1.11, –0.37 | <0.001 | –0.29 | –0.77, 0.19 | 0.231 | –0.64 | –1.09, –0.20 | 0.005 |  |  |  |  |  |  |  |  |  |  |  |  |
| Pubertal development [d] | –1.50 | –2.53, –0.48 | 0.004 | –1.99 | –3.33, –0.64 | 0.004 | –1.85 | –2.99, –0.70 | 0.002 |  |  |  |  |  |  |  |  |  |  |  |  |
| Socioeconomic position (z–score) | 0.24 | –0.15, 0.63 | 0.223 | 0.73 | 0.18, 1.27 | 0.010 | 0.7 | 0.25, 1.15 | 0.002 |  |  |  |  |  |  |  |  |  |  |  |  |
| School attendance on day of TUD (No) | –0.81 | –1.58, –0.03 | 0.041 | –1.25 | –2.30, –0.19 | 0.021 | –1.03 | –1.91, –0.15 | 0.022 |  |  |  |  |  |  |  |  |  |  |  |  |
| Season of measurement [e] | 0.28 | –0.06, 0.62 | 0.110 | 0.27 | –0.18, 0.72 | 0.245 | 0.25 | –0.13, 0.63 | 0.191 |  |  |  |  |  |  |  |  |  |  |  |  |
| PA = physical activity; HRQOL = health related quality of life, measured using PedsQL scales; β = model coefficient; CI = confidence interval. | | | | | | | | | | | | | | | | |  |  |  |  |  |
| a. The psychosocial HRQOL summary scale is a composite of social, emotional and school HRQOL | | | | | | | | | | | |  |  |  |  |  |  |  |  |  |  |
| b. The total HRQOL summary scale is a composite of physical, social, emotional and school HRQOL | | | | | | | | | | | |  |  |  |  |  |  |  |  |  |  |
| c. The coefficients presented in this table for this domain of PA represent the predicted effect associated with an increase of 30 min/day. | | | | | | | | | | | | | | | | | | | | | |
| d. The pubertal development scale ranged from 1 (least developed) to 4 (most developed). | | | | | | | | | | |  |  |  |  |  |  |  |  |  |  |  |
| e. Season of TUD completion was categorized as: 1= Winter; 2=Spring; 3=Summer; 4=Autumn. | | | | | | | | | | | |  |  |  |  |  |  |  |  |  |  |

**Table A2B: Associations between the absolute duration of organized PA at 10–11y and additional HRQOL outcomes at 10–11y**

|  | **Social HRQOL** | | | **Emotional HRQOL** | | | **School HRQOL** | | |  |  |  |  |  |  |  |  |  |  |  |  |
| --- | --- | --- | --- | --- | --- | --- | --- | --- | --- | --- | --- | --- | --- | --- | --- | --- | --- | --- | --- | --- | --- |
|  | **β** | **95% CI** | ***p*** | **β** | **95% CI** | ***p*** | **β** | **95% CI** | ***p*** |  |  |  |  |  |  |  |  |  |  |  |  |
| **Unadjusted models** |  |  |  |  |  |  |  |  |  |  |  |  |  |  |  |  |  |  |  |  |  |
| Organized PA (+30 min/day) [a] | 0.79 | 0.47, 1.12 | <0.001 | 0.61 | 0.29, 0.92 | <0.001 | 0.59 | 0.33, 0.84 | <0.001 |  |  |  |  |  |  |  |  |  |  |  |  |
| **Adjusted models** |  |  |  |  |  |  |  |  |  |  |  |  |  |  |  |  |  |  |  |  |  |
| Organized PA (+30 min/day) [a] | 0.65 | 0.32, 0.97 | <0.001 | 0.53 | 0.21, 0.84 | 0.001 | 0.46 | 0.21, 0.70 | <0.001 |  |  |  |  |  |  |  |  |  |  |  |  |
| Age (months) | 0.07 | –0.09, 0.22 | 0.388 | 0.06 | –0.09, 0.21 | 0.444 | –0.12 | –0.25, 0.02 | 0.088 |  |  |  |  |  |  |  |  |  |  |  |  |
| Sex (female) | –0.53 | –1.87, 0.81 | 0.438 | –0.47 | –1.83, 0.89 | 0.498 | 1.40 | 0.25, 2.56 | 0.017 |  |  |  |  |  |  |  |  |  |  |  |  |
| BMI (z–score) | –0.62 | –1.29, 0.06 | 0.073 | –0.03 | –0.49, 0.43 | 0.891 | –0.29 | –0.75, 0.16 | 0.205 |  |  |  |  |  |  |  |  |  |  |  |  |
| Pubertal development [b] | –2.38 | –3.89, –0.87 | 0.002 | –1.56 | –3.06, –0.05 | 0.043 | –1.51 | –2.78, –0.24 | 0.020 |  |  |  |  |  |  |  |  |  |  |  |  |
| Socioeconomic position (z–score) | 1.22 | 0.60, 1.85 | <0.001 | 0.14 | –0.47, 0.75 | 0.649 | 1.57 | 1.02, 2.12 | <0.001 |  |  |  |  |  |  |  |  |  |  |  |  |
| School attendance on day of TUD (No) | –1.11 | –2.30, 0.08 | 0.067 | –1.39 | –2.61, –0.18 | 0.024 | –1.01 | –2.04, 0.02 | 0.055 |  |  |  |  |  |  |  |  |  |  |  |  |
| Season of measurement [c] | 0.49 | –0.01, 1.00 | 0.056 | –0.01 | –0.52, +0.51 | 0.981 | 1.08 | 0.68, 1.49 | <0.001 |  |  |  |  |  |  |  |  |  |  |  |  |
| PA = physical activity; HRQOL = health related quality of life, measured using PedsQL scales; β = model coefficient; CI = confidence interval. | | | | | | | | | | | | | | | | |  |  |  |  |  |
| a. The coefficients presented in this table for this domain of PA represent the predicted effect associated with an increase of 30 min/day. | | | | | | | | | | | | | | | | | | | | | |
| b. The pubertal development scale ranged from 1 (least developed) to 4 (most developed). | | | | | | | | | | |  |  |  |  |  |  |  |  |  |  |  |
| c. Season of TUD completion was categorized as: 1= Winter; 2=Spring; 3=Summer; 4=Autumn. | | | | | | | | | | | |  |  |  |  |  |  |  |  |  |  |

**Table A3A: Associations between the absolute duration of active transport at 10–11y and main HRQOL outcomes at 10–11y**

|  | **Physical HRQOL** | | | **Psychosocial HRQOL [a]** | | | **Total HRQOL [b]** | | |  |  |  |  |  |  |  |  |  |  |  |  |
| --- | --- | --- | --- | --- | --- | --- | --- | --- | --- | --- | --- | --- | --- | --- | --- | --- | --- | --- | --- | --- | --- |
|  | **β** | **95% CI** | ***p*** | **β** | **95% CI** | ***p*** | **β** | **95% CI** | ***p*** |  |  |  |  |  |  |  |  |  |  |  |  |
| **Unadjusted models** |  |  |  |  |  |  |  |  |  |  |  |  |  |  |  |  |  |  |  |  |  |
| Active transport (+30 min/day) [c] | 0.14 | –0.22, 0.51 | 0.440 | 0.31 | –0.20, 0.82 | 0.236 | 0.19 | –0.24, 0.62 | 0.391 |  |  |  |  |  |  |  |  |  |  |  |  |
| **Adjusted models** |  |  |  |  |  |  |  |  |  |  |  |  |  |  |  |  |  |  |  |  |  |
| Active transport (+30 min/day) [c] | 0.05 | –0.29, 0.39 | 0.755 | 0.33 | –0.20, 0.86 | 0.220 | 0.21 | –0.21, 0.63 | 0.324 |  |  |  |  |  |  |  |  |  |  |  |  |
| Age (months) | 0.03 | –0.07, 0.13 | 0.551 | 0.07 | –0.07, 0.20 | 0.336 | 0.04 | –0.07, 0.16 | 0.444 |  |  |  |  |  |  |  |  |  |  |  |  |
| Sex (female) | –0.33 | –1.22, 0.55 | 0.457 | –0.63 | –1.83, 0.57 | 0.304 | –0.62 | –1.63, 0.40 | 0.234 |  |  |  |  |  |  |  |  |  |  |  |  |
| BMI (z–score) | –0.78 | –1.13, –0.43 | <0.001 | –0.34 | –0.81, 0.12 | 0.145 | –0.70 | –1.12, –0.27 | 0.001 |  |  |  |  |  |  |  |  |  |  |  |  |
| Pubertal development [d] | –1.57 | –2.60, –0.54 | 0.003 | –2.06 | –3.41, –0.72 | 0.003 | –1.91 | –3.06, –0.76 | 0.001 |  |  |  |  |  |  |  |  |  |  |  |  |
| Socioeconomic position (z–score) | 0.31 | –0.08, 0.70 | 0.115 | 0.79 | 0.24, 1.34 | 0.005 | 0.77 | 0.32, 1.22 | 0.001 |  |  |  |  |  |  |  |  |  |  |  |  |
| School attendance on day of TUD (No) | –0.95 | –1.73, –0.18 | 0.016 | –1.38 | –2.44, –0.32 | 0.011 | –1.18 | –2.06, –0.30 | 0.009 |  |  |  |  |  |  |  |  |  |  |  |  |
| Season of measurement [e] | 0.25 | –0.09, 0.59 | 0.145 | 0.25 | –0.20, 0.69 | 0.285 | 0.22 | –0.15, 0.60 | 0.242 |  |  |  |  |  |  |  |  |  |  |  |  |
| PA = physical activity; HRQOL = health related quality of life, measured using PedsQL scales; β = model coefficient; CI = confidence interval. | | | | | | | | | | | | | | | | |  |  |  |  |  |
| a. The psychosocial HRQOL summary scale is a composite of social, emotional and school HRQOL | | | | | | | | | | | |  |  |  |  |  |  |  |  |  |  |
| b. The total HRQOL summary scale is a composite of physical, social, emotional and school HRQOL | | | | | | | | | | | |  |  |  |  |  |  |  |  |  |  |
| c. The coefficients presented in this table for this domain of PA represent the predicted effect associated with an increase of 30 min/day. | | | | | | | | | | | | | | | | | | | | | |
| d. The pubertal development scale ranged from 1 (least developed) to 4 (most developed). | | | | | | | | | | |  |  |  |  |  |  |  |  |  |  |  |
| e. Season of TUD completion was categorized as: 1= Winter; 2=Spring; 3=Summer; 4=Autumn. | | | | | | | | | | | |  |  |  |  |  |  |  |  |  |  |

**Table A3B: Associations between the absolute duration of active transport at 10–11y and additional HRQOL outcomes at 10–11y**

|  | **Social HRQOL** | | | **Emotional HRQOL** | | | **School HRQOL** | | |  |  |  |  |  |  |  |  |  |  |  |  |
| --- | --- | --- | --- | --- | --- | --- | --- | --- | --- | --- | --- | --- | --- | --- | --- | --- | --- | --- | --- | --- | --- |
|  | **β** | **95% CI** | ***p*** | **β** | **95% CI** | ***p*** | **β** | **95% CI** | ***p*** |  |  |  |  |  |  |  |  |  |  |  |  |
| **Unadjusted models** |  |  |  |  |  |  |  |  |  |  |  |  |  |  |  |  |  |  |  |  |  |
| Active transport (+30 min/day) [a] | 0.32 | –0.46, 1.10 | 0.424 | 0.34 | –0.29, 0.98 | 0.289 | 0.67 | 0.22, 1.12 | 0.004 |  |  |  |  |  |  |  |  |  |  |  |  |
| **Adjusted models** |  |  |  |  |  |  |  |  |  |  |  |  |  |  |  |  |  |  |  |  |  |
| Active transport (+30 min/day) [a] | 0.40 | –0.40, 1.19 | 0.329 | 0.36 | –0.33, 1.04 | 0.307 | 0.70 | 0.26, 1.13 | 0.002 |  |  |  |  |  |  |  |  |  |  |  |  |
| Age (months) | 0.06 | –0.10, 0.21 | 0.459 | 0.05 | –0.10, 0.21 | 0.497 | –0.12 | –0.26, 0.01 | 0.071 |  |  |  |  |  |  |  |  |  |  |  |  |
| Sex (female) | –0.55 | –1.89, 0.80 | 0.426 | –0.48 | –1.84, 0.88 | 0.490 | 1.36 | 0.20, 2.51 | 0.021 |  |  |  |  |  |  |  |  |  |  |  |  |
| BMI (z–score) | –0.65 | –1.32, 0.01 | 0.055 | –0.08 | –0.55, 0.38 | 0.728 | –0.33 | –0.77, 0.11 | 0.141 |  |  |  |  |  |  |  |  |  |  |  |  |
| Pubertal development [b] | –2.47 | –3.98, –0.96 | 0.001 | –1.64 | –3.15, –0.13 | 0.033 | –1.51 | –2.79, –0.24 | 0.020 |  |  |  |  |  |  |  |  |  |  |  |  |
| Socioeconomic position (z–score) | 1.31 | 0.69, 1.94 | <0.001 | 0.21 | –0.41, 0.82 | 0.511 | 1.63 | 1.08, 2.19 | <0.001 |  |  |  |  |  |  |  |  |  |  |  |  |
| School attendance on day of TUD (No) | –1.23 | –2.43, –0.03 | 0.044 | –1.51 | –2.72, –0.29 | 0.015 | –1.09 | –2.12, –0.06 | 0.038 |  |  |  |  |  |  |  |  |  |  |  |  |
| Season of measurement [c] | 0.47 | –0.04, 0.98 | 0.070 | –0.03 | –0.55, 0.48 | 0.903 | 1.07 | 0.66, 1.47 | <0.001 |  |  |  |  |  |  |  |  |  |  |  |  |
| PA = physical activity; HRQOL = health related quality of life, measured using PedsQL scales; β = model coefficient; CI = confidence interval. | | | | | | | | | | | | | | | | |  |  |  |  |  |
| a. The coefficients presented in this table for this domain of PA represent the predicted effect associated with an increase of 30 min/day. | | | | | | | | | | | | | | | | | | | | | |
| b. The pubertal development scale ranged from 1 (least developed) to 4 (most developed). | | | | | | | | | | |  |  |  |  |  |  |  |  |  |  |  |
| c. Season of TUD completion was categorized as: 1= Winter; 2=Spring; 3=Summer; 4=Autumn. | | | | | | | | | | | |  |  |  |  |  |  |  |  |  |  |

**Table A4A: Associations between the absolute duration of active chores/work at 10–11y and main HRQOL outcomes at 10–11y**

|  | **Physical HRQOL** | | | **Psychosocial HRQOL [a]** | | | **Total HRQOL [b]** | | |  |  |  |  |  |  |  |  |  |  |  |  |
| --- | --- | --- | --- | --- | --- | --- | --- | --- | --- | --- | --- | --- | --- | --- | --- | --- | --- | --- | --- | --- | --- |
|  | **β** | **95% CI** | ***p*** | **β** | **95% CI** | ***p*** | **β** | **95% CI** | ***p*** |  |  |  |  |  |  |  |  |  |  |  |  |
| **Unadjusted models** |  |  |  |  |  |  |  |  |  |  |  |  |  |  |  |  |  |  |  |  |  |
| Active chores/work (+30 min/day) [c] | –0.05 | –0.33, 0.23 | 0.724 | –0.26 | –0.65, 0.14 | 0.206 | –0.15 | –0.46, 0.17 | 0.359 |  |  |  |  |  |  |  |  |  |  |  |  |
| **Adjusted models** |  |  |  |  |  |  |  |  |  |  |  |  |  |  |  |  |  |  |  |  |  |
| Active chores/work (+30 min/day) [c] | –0.01 | –0.31, 0.29 | 0.951 | –0.15 | –0.57, 0.27 | 0.474 | –0.07 | –0.41, 0.26 | 0.670 |  |  |  |  |  |  |  |  |  |  |  |  |
| Age (months) | 0.03 | –0.07, 0.13 | 0.543 | 0.07 | –0.06, 0.21 | 0.304 | 0.05 | –0.07, 0.16 | 0.415 |  |  |  |  |  |  |  |  |  |  |  |  |
| Sex (female) | –0.33 | –1.21, 0.55 | 0.463 | –0.60 | –1.81, 0.60 | 0.329 | –0.60 | –1.62, 0.42 | 0.248 |  |  |  |  |  |  |  |  |  |  |  |  |
| BMI (z–score) | –0.78 | –1.13, –0.43 | <0.001 | –0.34 | –0.80, 0.12 | 0.148 | –0.70 | –1.12, –0.27 | 0.001 |  |  |  |  |  |  |  |  |  |  |  |  |
| Pubertal development [d] | –1.58 | –2.60, –0.55 | 0.003 | –2.08 | –3.42, –0.73 | 0.002 | –1.93 | –3.07, –0.78 | 0.001 |  |  |  |  |  |  |  |  |  |  |  |  |
| Socioeconomic position (z–score) | 0.31 | –0.08, 0.70 | 0.115 | 0.80 | 0.25, 1.35 | 0.004 | 0.78 | 0.33, 1.22 | 0.001 |  |  |  |  |  |  |  |  |  |  |  |  |
| School attendance on day of TUD (No) | –0.95 | –1.75, –0.16 | 0.019 | –1.33 | –2.41, –0.25 | 0.016 | –1.16 | –2.06, –0.25 | 0.012 |  |  |  |  |  |  |  |  |  |  |  |  |
| Season of measurement [e] | 0.25 | –0.09, 0.59 | 0.145 | 0.25 | –0.20, 0.70 | 0.275 | 0.23 | –0.15, 0.60 | 0.235 |  |  |  |  |  |  |  |  |  |  |  |  |
| PA = physical activity; HRQOL = health related quality of life, measured using PedsQL scales; β = model coefficient; CI = confidence interval. | | | | | | | | | | | | | | | | |  |  |  |  |  |
| a. The psychosocial HRQOL summary scale is a composite of social, emotional and school HRQOL | | | | | | | | | | | |  |  |  |  |  |  |  |  |  |  |
| b. The total HRQOL summary scale is a composite of physical, social, emotional and school HRQOL | | | | | | | | | | | |  |  |  |  |  |  |  |  |  |  |
| c. The coefficients presented in this table for this domain of PA represent the predicted effect associated with an increase of 30 min/day. | | | | | | | | | | | | | | | | | | | | | |
| d. The pubertal development scale ranged from 1 (least developed) to 4 (most developed). | | | | | | | | | | |  |  |  |  |  |  |  |  |  |  |  |
| e. Season of TUD completion was categorized as: 1= Winter; 2=Spring; 3=Summer; 4=Autumn. | | | | | | | | | | | |  |  |  |  |  |  |  |  |  |  |

**Table A4B: Associations between the absolute duration of active chores/work at 10–11y and additional HRQOL outcomes at 10–11y**

|  | **Social HRQOL** | | | **Emotional HRQOL** | | | **School HRQOL** | | |  |  |  |  |  |  |  |  |  |  |  |  |
| --- | --- | --- | --- | --- | --- | --- | --- | --- | --- | --- | --- | --- | --- | --- | --- | --- | --- | --- | --- | --- | --- |
|  | **β** | **95% CI** | ***p*** | **β** | **95% CI** | ***p*** | **β** | **95% CI** | ***p*** |  |  |  |  |  |  |  |  |  |  |  |  |
| **Unadjusted models** |  |  |  |  |  |  |  |  |  |  |  |  |  |  |  |  |  |  |  |  |  |
| Active chores/work (+30 min/day) [a] | –0.26 | –0.73, 0.22 | 0.296 | –0.23 | –0.67, 0.21 | 0.304 | –0.13 | –0.50, 0.25 | 0.503 |  |  |  |  |  |  |  |  |  |  |  |  |
| **Adjusted models** |  |  |  |  |  |  |  |  |  |  |  |  |  |  |  |  |  |  |  |  |  |
| Active chores/work (+30 min/day) [a] | –0.14 | –0.61, 0.33 | 0.555 | –0.16 | –0.64, 0.32 | 0.511 | –0.04 | –0.41, 0.33 | 0.830 |  |  |  |  |  |  |  |  |  |  |  |  |
| Age (months) | 0.06 | –0.09, 0.22 | 0.420 | 0.06 | –0.10, 0.21 | 0.464 | –0.12 | –0.25, 0.01 | 0.079 |  |  |  |  |  |  |  |  |  |  |  |  |
| Sex (female) | –0.53 | –1.87, 0.82 | 0.445 | –0.45 | –1.81, 0.92 | 0.521 | 1.36 | 0.20, 2.52 | 0.021 |  |  |  |  |  |  |  |  |  |  |  |  |
| BMI (z–score) | –0.64 | –1.31, 0.02 | 0.057 | –0.08 | –0.55, 0.38 | 0.735 | –0.33 | –0.77, 0.11 | 0.144 |  |  |  |  |  |  |  |  |  |  |  |  |
| Pubertal development [b] | –2.49 | –4.01, –0.98 | 0.001 | –1.65 | –3.16, –0.14 | 0.032 | –1.54 | –2.82, –0.27 | 0.017 |  |  |  |  |  |  |  |  |  |  |  |  |
| Socioeconomic position (z–score) | 1.31 | 0.69, 1.94 | <0.001 | 0.21 | –0.40, 0.82 | 0.506 | 1.64 | 1.08, 2.19 | <0.001 |  |  |  |  |  |  |  |  |  |  |  |  |
| School attendance on day of TUD (No) | –1.21 | –2.43, 0.01 | 0.053 | –1.45 | –2.69, –0.22 | 0.021 | –1.12 | –2.19, –0.06 | 0.038 |  |  |  |  |  |  |  |  |  |  |  |  |
| Season of measurement [c] | 0.48 | –0.03, 0.99 | 0.067 | –0.03 | –0.54, 0.49 | 0.917 | 1.06 | 0.66, 1.47 | <0.001 |  |  |  |  |  |  |  |  |  |  |  |  |
| PA = physical activity; HRQOL = health related quality of life, measured using PedsQL scales; β = model coefficient; CI = confidence interval. | | | | | | | | | | | | | | | | |  |  |  |  |  |
| a. The coefficients presented in this table for this domain of PA represent the predicted effect associated with an increase of 30 min/day. | | | | | | | | | | | | | | | | | | | | | |
| b. The pubertal development scale ranged from 1 (least developed) to 4 (most developed). | | | | | | | | | | |  |  |  |  |  |  |  |  |  |  |  |
| c. Season of TUD completion was categorized as: 1= Winter; 2=Spring; 3=Summer; 4=Autumn. | | | | | | | | | | | |  |  |  |  |  |  |  |  |  |  |

***Longitudinal models***

**Table A5A: Associations between the absolute duration of non–organized PA at 10–11y and main HRQOL outcomes at 12–13y**

|  | **Physical HRQOL** | | | **Psychosocial HRQOL [a]** | | | **Total HRQOL [b]** | | |  |  |  |  |  |  |  |  |  |  |  |  |
| --- | --- | --- | --- | --- | --- | --- | --- | --- | --- | --- | --- | --- | --- | --- | --- | --- | --- | --- | --- | --- | --- |
|  | **β** | **95% CI** | ***p*** | **β** | **95% CI** | ***p*** | **β** | **95% CI** | ***p*** |  |  |  |  |  |  |  |  |  |  |  |  |
| **Unadjusted models** |  |  |  |  |  |  |  |  |  |  |  |  |  |  |  |  |  |  |  |  |  |
| Non–organized PA (+30 min/day) [c] | 0.18 | 0.02, 0.33 | 0.023 | 0.34 | 0.14, 0.53 | 0.001 | 0.28 | 0.11, 0.44 | 0.001 |  |  |  |  |  |  |  |  |  |  |  |  |
| **Adjusted models** |  |  |  |  |  |  |  |  |  |  |  |  |  |  |  |  |  |  |  |  |  |
| Non–organized PA (+30 min/day) [c] | 0.01 | –0.10, 0.13 | 0.832 | 0.17 | 0.03, 0.32 | 0.021 | 0.10 | –0.02, 0.22 | 0.119 |  |  |  |  |  |  |  |  |  |  |  |  |
| Age (months) | 0.00 | –0.10, 0.09 | 0.979 | 0.00 | –0.11, 0.11 | 0.972 | –0.01 | –0.10, 0.09 | 0.903 |  |  |  |  |  |  |  |  |  |  |  |  |
| Sex (female) | –0.18 | –0.97, 0.61 | 0.656 | –0.76 | –1.71, 0.19 | 0.115 | –0.37 | –1.16, 0.42 | 0.359 |  |  |  |  |  |  |  |  |  |  |  |  |
| BMI (z–score) | –0.13 | –0.45, 0.19 | 0.422 | –0.07 | –0.52, 0.38 | 0.761 | –0.03 | –0.35, 0.30 | 0.868 |  |  |  |  |  |  |  |  |  |  |  |  |
| Pubertal development [d] | –0.75 | –1.64, 0.13 | 0.097 | –0.65 | –1.67, 0.38 | 0.215 | –0.93 | –1.78, –0.09 | 0.031 |  |  |  |  |  |  |  |  |  |  |  |  |
| Socioeconomic position (z–score) | 0.69 | 0.33, 1.05 | <0.001 | 0.67 | 0.24, 1.09 | 0.002 | 0.75 | 0.39, 1.10 | <0.001 |  |  |  |  |  |  |  |  |  |  |  |  |
| School attendance on day of TUD (No) | 0.23 | –0.47, 0.94 | 0.518 | 0.04 | –0.81, 0.89 | 0.931 | 0.26 | –0.43, 0.95 | 0.466 |  |  |  |  |  |  |  |  |  |  |  |  |
| Season of measurement [e] | 0.07 | –0.23, 0.36 | 0.647 | –0.06 | –0.42, 0.29 | 0.727 | –0.02 | –0.31, 0.27 | 0.880 |  |  |  |  |  |  |  |  |  |  |  |  |
| PedsQL outcome at 10–11y [f] | 0.64 | 0.58, 0.70 | <0.001 | 0.67 | 0.64, 0.70 | <0.001 | 0.71 | 0.67, 0.75 | <0.001 |  |  |  |  |  |  |  |  |  |  |  |  |
| PA = physical activity; HRQOL = health related quality of life, measured using PedsQL scales; β = model coefficient; CI = confidence interval. | | | | | | | | | | | | | | | | |  |  |  |  |  |
| a. The psychosocial HRQOL summary scale is a composite of social, emotional and school HRQOL | | | | | | | | | | | |  |  |  |  |  |  |  |  |  |  |
| b. The total HRQOL summary scale is a composite of physical, social, emotional and school HRQOL | | | | | | | | | | | |  |  |  |  |  |  |  |  |  |  |
| c. The coefficients presented in this table for this domain of PA represent the predicted effect associated with an increase of 30 min/day. | | | | | | | | | | | | | | | | | | | | | |
| d. The pubertal development scale ranged from 1 (least developed) to 4 (most developed). | | | | | | | | | | |  |  |  |  |  |  |  |  |  |  |  |
| e. Season of TUD completion was categorized as: 1= Winter; 2=Spring; 3=Summer; 4=Autumn. | | | | | | | | | | | |  |  |  |  |  |  |  |  |  |  |
| f. This variable refers to the corresponding PedsQL domain measured at 10–11y (e.g. the PedsQL physical scale at 10–11y was included in the physical HRQOL model, and so forth). | | | | | | | | | | | | | | | |  |  |  |  |  |  |

**Table A5B: Associations between the absolute duration of non–organized PA at 10–11y and additional HRQOL outcomes at 12–13y**

|  | **Social HRQOL** | | | **Emotional HRQOL** | | | **School HRQOL** | | |  |  |  |  |  |  |  |  |  |  |  |  |
| --- | --- | --- | --- | --- | --- | --- | --- | --- | --- | --- | --- | --- | --- | --- | --- | --- | --- | --- | --- | --- | --- |
|  | **β** | **95% CI** | ***p*** | **β** | **95% CI** | ***p*** | **β** | **95% CI** | ***p*** |  |  |  |  |  |  |  |  |  |  |  |  |
| **Unadjusted models** |  |  |  |  |  |  |  |  |  |  |  |  |  |  |  |  |  |  |  |  |  |
| Non–organized PA (+30 min/day) [a] | 0.33 | 0.12, 0.54 | 0.002 | 0.32 | 0.10, 0.53 | 0.004 | –0.01 | –0.22, 0.20 | 0.899 |  |  |  |  |  |  |  |  |  |  |  |  |
| **Adjusted models** |  |  |  |  |  |  |  |  |  |  |  |  |  |  |  |  |  |  |  |  |  |
| Non–organized PA (+30 min/day) [a] | 0.16 | –0.02, 0.33 | 0.082 | 0.16 | –0.01, 0.34 | 0.064 | –0.09 | –0.30, 0.12 | 0.397 |  |  |  |  |  |  |  |  |  |  |  |  |
| Age (months) | –0.09 | –0.22, 0.04 | 0.182 | 0.10 | –0.03, 0.23 | 0.120 | –0.05 | –0.20, 0.11 | 0.562 |  |  |  |  |  |  |  |  |  |  |  |  |
| Sex (female) | 0.20 | –0.90, 1.31 | 0.716 | –1.97 | –3.08, –0.86 | 0.001 | 1.12 | –0.12, 2.37 | 0.077 |  |  |  |  |  |  |  |  |  |  |  |  |
| BMI (z–score) | –0.59 | –1.03, –0.15 | 0.009 | 0.45 | –0.08, 0.97 | 0.095 | –0.27 | –0.70, 0.16 | 0.216 |  |  |  |  |  |  |  |  |  |  |  |  |
| Pubertal development [b] | –0.24 | –1.42, 0.93 | 0.683 | –0.80 | –2.01, 0.40 | 0.191 | –1.56 | –2.93, –0.18 | 0.026 |  |  |  |  |  |  |  |  |  |  |  |  |
| Socioeconomic position (z–score) | 1.07 | 0.57, 1.57 | <0.001 | 0.47 | –0.04, 0.98 | 0.070 | 1.63 | 1.01, 2.26 | <0.001 |  |  |  |  |  |  |  |  |  |  |  |  |
| School attendance on day of TUD (No) | 0.51 | –0.49, 1.50 | 0.317 | –0.48 | –1.46, 0.50 | 0.340 | –0.51 | –1.67, 0.65 | 0.392 |  |  |  |  |  |  |  |  |  |  |  |  |
| Season of measurement [c] | –0.07 | –0.49, 0.35 | 0.751 | –0.11 | –0.53, 0.31 | 0.610 | –0.03 | –0.51, 0.45 | 0.902 |  |  |  |  |  |  |  |  |  |  |  |  |
| PedsQL outcome at 10–11y [d] | 0.61 | 0.56, 0.65 | <0.001 | 0.61 | 0.58, 0.65 | <0.001 | 0.26 | 0.21, 0.31 | <0.001 |  |  |  |  |  |  |  |  |  |  |  |  |
| PA = physical activity; HRQOL = health related quality of life, measured using PedsQL scales; β = model coefficient; CI = confidence interval. | | | | | | | | | | | | | | | | |  |  |  |  |  |
| a. The coefficients presented in this table for this domain of PA represent the predicted effect associated with an increase of 30 min/day. | | | | | | | | | | | | | | | | | | | | | |
| b. The pubertal development scale ranged from 1 (least developed) to 4 (most developed). | | | | | | | | | | |  |  |  |  |  |  |  |  |  |  |  |
| c. Season of TUD completion was categorized as: 1= Winter; 2=Spring; 3=Summer; 4=Autumn. | | | | | | | | | | | |  |  |  |  |  |  |  |  |  |  |
| d. This variable refers to the corresponding PedsQL domain measured at 10–11y (e.g. the PedsQL physical scale at 10–11y was included in the physical HRQOL model, and so forth). | | | | | | | | | | | | | | | |  |  |  |  |  |  |

**Table A6A: Associations between the absolute duration of organized PA at 10–11y and main HRQOL outcomes at 12–13y**

|  | **Physical HRQOL** | | | **Psychosocial HRQOL [a]** | | | **Total HRQOL [b]** | | |  |  |  |  |  |  |  |  |  |  |  |  |
| --- | --- | --- | --- | --- | --- | --- | --- | --- | --- | --- | --- | --- | --- | --- | --- | --- | --- | --- | --- | --- | --- |
|  | **β** | **95% CI** | ***p*** | **β** | **95% CI** | ***p*** | **β** | **95% CI** | ***p*** |  |  |  |  |  |  |  |  |  |  |  |  |
| **Unadjusted models** |  |  |  |  |  |  |  |  |  |  |  |  |  |  |  |  |  |  |  |  |  |
| Organized PA (+30 min/day) [c] | 0.39 | 0.17, 0.61 | <0.001 | 0.68 | 0.41, 0.95 | <0.001 | 0.61 | 0.38, 0.84 | <0.001 |  |  |  |  |  |  |  |  |  |  |  |  |
| **Adjusted models** |  |  |  |  |  |  |  |  |  |  |  |  |  |  |  |  |  |  |  |  |  |
| Organized PA (+30 min/day) [c] | 0.04 | –0.11, 0.20 | 0.577 | 0.21 | 0.00, 0.41 | 0.054 | 0.12 | –0.05, 0.28 | 0.174 |  |  |  |  |  |  |  |  |  |  |  |  |
| Age (months) | 0.00 | –0.10, 0.09 | 0.989 | 0.00 | –0.11, 0.12 | 0.957 | 0.00 | –0.10, 0.09 | 0.957 |  |  |  |  |  |  |  |  |  |  |  |  |
| Sex (female) | –0.19 | –0.98, 0.60 | 0.642 | –0.84 | –1.79, 0.10 | 0.081 | –0.41 | –1.20, 0.37 | 0.301 |  |  |  |  |  |  |  |  |  |  |  |  |
| BMI (z–score) | –0.13 | –0.45, 0.19 | 0.442 | –0.08 | –0.50, 0.34 | 0.710 | –0.02 | –0.34, 0.30 | 0.884 |  |  |  |  |  |  |  |  |  |  |  |  |
| Pubertal development [d] | –0.75 | –1.64, 0.14 | 0.098 | –0.64 | –1.66, 0.38 | 0.220 | –0.93 | –1.78, –0.09 | 0.031 |  |  |  |  |  |  |  |  |  |  |  |  |
| Socioeconomic position (z–score) | 0.69 | 0.33, 1.04 | <0.001 | 0.62 | 0.20, 1.04 | 0.004 | 0.73 | 0.37, 1.08 | <0.001 |  |  |  |  |  |  |  |  |  |  |  |  |
| School attendance on day of TUD (No) | 0.26 | –0.44, 0.95 | 0.468 | 0.25 | –0.59, 1.09 | 0.563 | 0.37 | –0.31, 1.05 | 0.287 |  |  |  |  |  |  |  |  |  |  |  |  |
| Season of measurement [e] | 0.07 | –0.22, 0.37 | 0.632 | –0.04 | –0.39, 0.32 | 0.834 | –0.01 | –0.30, 0.28 | 0.955 |  |  |  |  |  |  |  |  |  |  |  |  |
| PedsQL outcome at 10–11y [f] | 0.64 | 0.58, 0.70 | <0.001 | 0.67 | 0.63, 0.70 | <0.001 | 0.71 | 0.67, 0.75 | <0.001 |  |  |  |  |  |  |  |  |  |  |  |  |
| PA = physical activity; HRQOL = health related quality of life, measured using PedsQL scales; β = model coefficient; CI = confidence interval. | | | | | | | | | | | | | | | | |  |  |  |  |  |
| a. The psychosocial HRQOL summary scale is a composite of social, emotional and school HRQOL | | | | | | | | | | | |  |  |  |  |  |  |  |  |  |  |
| b. The total HRQOL summary scale is a composite of physical, social, emotional and school HRQOL | | | | | | | | | | | |  |  |  |  |  |  |  |  |  |  |
| c. The coefficients presented in this table for this domain of PA represent the predicted effect associated with an increase of 30 min/day. | | | | | | | | | | | | | | | | | | | | | |
| d. The pubertal development scale ranged from 1 (least developed) to 4 (most developed). | | | | | | | | | | |  |  |  |  |  |  |  |  |  |  |  |
| e. Season of TUD completion was categorized as: 1= Winter; 2=Spring; 3=Summer; 4=Autumn. | | | | | | | | | | | |  |  |  |  |  |  |  |  |  |  |
| f. This variable refers to the corresponding PedsQL domain measured at 10–11y (e.g. the PedsQL physical scale at 10–11y was included in the physical HRQOL model, and so forth). | | | | | | | | | | | | | | | |  |  |  |  |  |  |

**Table A6B: Associations between the absolute duration of organized PA at 10–11y and additional HRQOL outcomes at 12–13y**

|  | **Social HRQOL** | | | **Emotional HRQOL** | | | **School HRQOL** | | |  |  |  |  |  |  |  |  |  |  |  |  |
| --- | --- | --- | --- | --- | --- | --- | --- | --- | --- | --- | --- | --- | --- | --- | --- | --- | --- | --- | --- | --- | --- |
|  | **β** | **95% CI** | ***p*** | **β** | **95% CI** | ***p*** | **β** | **95% CI** | ***p*** |  |  |  |  |  |  |  |  |  |  |  |  |
| **Unadjusted models** |  |  |  |  |  |  |  |  |  |  |  |  |  |  |  |  |  |  |  |  |  |
| Organized PA (+30 min/day) [a] | 0.58 | 0.29, 0.88 | <0.001 | 0.73 | 0.42, 1.04 | <0.001 | 0.41 | 0.13, 0.70 | 0.005 |  |  |  |  |  |  |  |  |  |  |  |  |
| **Adjusted models** |  |  |  |  |  |  |  |  |  |  |  |  |  |  |  |  |  |  |  |  |  |
| Organized PA (+30 min/day) [a] | 0.18 | –0.06, 0.42 | 0.144 | 0.26 | 0.02, 0.50 | 0.034 | –0.02 | –0.30, 0.27 | 0.903 |  |  |  |  |  |  |  |  |  |  |  |  |
| Age (months) | –0.08 | –0.22, 0.05 | 0.213 | 0.11 | –0.02, 0.23 | 0.112 | 0.04 | –0.10, 0.18 | 0.592 |  |  |  |  |  |  |  |  |  |  |  |  |
| Sex (female) | 0.14 | –0.96, 1.24 | 0.799 | –2.06 | –3.17, –0.95 | <0.001 | 0.79 | –0.38, 1.95 | 0.187 |  |  |  |  |  |  |  |  |  |  |  |  |
| BMI (z–score) | –0.59 | –1.01, –0.17 | 0.006 | 0.45 | –0.06, 0.96 | 0.082 | –0.33 | –0.72, 0.07 | 0.104 |  |  |  |  |  |  |  |  |  |  |  |  |
| Pubertal development [b] | –0.25 | –1.42, 0.92 | 0.676 | –0.77 | –1.98, 0.44 | 0.213 | –1.29 | –2.57, –0.01 | 0.049 |  |  |  |  |  |  |  |  |  |  |  |  |
| Socioeconomic position (z–score) | 1.03 | 0.53, 1.54 | <0.001 | 0.42 | –0.09, 0.94 | 0.103 | 1.05 | 0.44, 1.65 | 0.001 |  |  |  |  |  |  |  |  |  |  |  |  |
| School attendance on day of TUD (No) | 0.71 | –0.28, 1.69 | 0.161 | –0.27 | –1.25, 0.71 | 0.587 | –0.16 | –1.23, 0.91 | 0.768 |  |  |  |  |  |  |  |  |  |  |  |  |
| Season of measurement [c] | –0.05 | –0.47, 0.37 | 0.818 | –0.08 | –0.50, 0.34 | 0.716 | –0.30 | –0.76, 0.15 | 0.189 |  |  |  |  |  |  |  |  |  |  |  |  |
| PedsQL outcome at 10–11y [d] | 0.61 | 0.56, 0.65 | <0.001 | 0.61 | 0.58, 0.65 | <0.001 | 0.42 | 0.38, 0.47 | <0.001 |  |  |  |  |  |  |  |  |  |  |  |  |
| PA = physical activity; HRQOL = health related quality of life, measured using PedsQL scales; β = model coefficient; CI = confidence interval. | | | | | | | | | | | | | | | | |  |  |  |  |  |
| a. The coefficients presented in this table for this domain of PA represent the predicted effect associated with an increase of 30 min/day. | | | | | | | | | | | | | | | | | | | | | |
| b. The pubertal development scale ranged from 1 (least developed) to 4 (most developed). | | | | | | | | | | |  |  |  |  |  |  |  |  |  |  |  |
| c. Season of TUD completion was categorized as: 1= Winter; 2=Spring; 3=Summer; 4=Autumn. | | | | | | | | | | | |  |  |  |  |  |  |  |  |  |  |
| d. This variable refers to the corresponding PedsQL domain measured at 10–11y (e.g. the PedsQL physical scale at 10–11y was included in the physical HRQOL model, and so forth). | | | | | | | | | | | | | | | |  |  |  |  |  |  |

**Table A7A: Associations between the absolute duration of active transport at 10–11y and main HRQOL outcomes at 12–13y**

|  | **Physical HRQOL** | | | **Psychosocial HRQOL [a]** | | | **Total HRQOL [b]** | | |  |  |  |  |  |  |  |  |  |  |  |  |
| --- | --- | --- | --- | --- | --- | --- | --- | --- | --- | --- | --- | --- | --- | --- | --- | --- | --- | --- | --- | --- | --- |
|  | **β** | **95% CI** | ***p*** | **β** | **95% CI** | ***p*** | **β** | **95% CI** | ***p*** |  |  |  |  |  |  |  |  |  |  |  |  |
| **Unadjusted models** |  |  |  |  |  |  |  |  |  |  |  |  |  |  |  |  |  |  |  |  |  |
| Active transport (+30 min/day) [c] | –0.32 | –0.89, 0.25 | 0.264 | 0.14 | –0.44, 0.73 | 0.637 | –0.06 | –0.57, 0.45 | 0.825 |  |  |  |  |  |  |  |  |  |  |  |  |
| **Adjusted models** |  |  |  |  |  |  |  |  |  |  |  |  |  |  |  |  |  |  |  |  |  |
| Active transport (+30 min/day) [c] | –0.29 | –0.70, 0.11 | 0.157 | 0.05 | –0.37, 0.47 | 0.806 | –0.12 | –0.46, 0.23 | 0.503 |  |  |  |  |  |  |  |  |  |  |  |  |
| Age (months) | 0.00 | –0.09, 0.10 | 0.957 | 0.00 | –0.11, 0.11 | 0.978 | 0.00 | –0.10, 0.09 | 0.960 |  |  |  |  |  |  |  |  |  |  |  |  |
| Sex (female) | –0.19 | –0.98, 0.60 | 0.638 | –0.84 | –1.78, 0.11 | 0.084 | –0.42 | –1.20, 0.37 | 0.300 |  |  |  |  |  |  |  |  |  |  |  |  |
| BMI (z–score) | –0.13 | –0.44, 0.19 | 0.439 | –0.08 | –0.52, 0.36 | 0.707 | –0.03 | –0.35, 0.30 | 0.868 |  |  |  |  |  |  |  |  |  |  |  |  |
| Pubertal development [d] | –0.77 | –1.66, 0.11 | 0.088 | –0.67 | –1.70, 0.35 | 0.197 | –0.95 | –1.80, –0.10 | 0.028 |  |  |  |  |  |  |  |  |  |  |  |  |
| Socioeconomic position (z–score) | 0.70 | 0.34, 1.06 | <0.001 | 0.65 | 0.22, 1.07 | 0.003 | 0.74 | 0.39, 1.10 | <0.001 |  |  |  |  |  |  |  |  |  |  |  |  |
| School attendance on day of TUD (No) | 0.22 | –0.48, 0.91 | 0.544 | 0.19 | –0.65, 1.03 | 0.665 | 0.32 | –0.36, 1.01 | 0.353 |  |  |  |  |  |  |  |  |  |  |  |  |
| Season of measurement [e] | 0.07 | –0.23, 0.36 | 0.648 | –0.05 | –0.40, 0.31 | 0.804 | –0.01 | –0.30, 0.27 | 0.920 |  |  |  |  |  |  |  |  |  |  |  |  |
| PedsQL outcome at 10–11y [f] | 0.64 | 0.58, 0.70 | <0.001 | 0.67 | 0.64, 0.70 | <0.001 | 0.71 | 0.67, 0.75 | <0.001 |  |  |  |  |  |  |  |  |  |  |  |  |
| PA = physical activity; HRQOL = health related quality of life, measured using PedsQL scales; β = model coefficient; CI = confidence interval. | | | | | | | | | | | | | | | | |  |  |  |  |  |
| a. The psychosocial HRQOL summary scale is a composite of social, emotional and school HRQOL | | | | | | | | | | | |  |  |  |  |  |  |  |  |  |  |
| b. The total HRQOL summary scale is a composite of physical, social, emotional and school HRQOL | | | | | | | | | | | |  |  |  |  |  |  |  |  |  |  |
| c. The coefficients presented in this table for this domain of PA represent the predicted effect associated with an increase of 30 min/day. | | | | | | | | | | | | | | | | | | | | | |
| d. The pubertal development scale ranged from 1 (least developed) to 4 (most developed). | | | | | | | | | | |  |  |  |  |  |  |  |  |  |  |  |
| e. Season of TUD completion was categorized as: 1= Winter; 2=Spring; 3=Summer; 4=Autumn. | | | | | | | | | | | |  |  |  |  |  |  |  |  |  |  |
| f. This variable refers to the corresponding PedsQL domain measured at 10–11y (e.g. the PedsQL physical scale at 10–11y was included in the physical HRQOL model, and so forth). | | | | | | | | | | | | | | | |  |  |  |  |  |  |

**Table A7B: Associations between the absolute duration of active transport at 10–11y and additional HRQOL outcomes at 12–13y**

|  | **Social HRQOL** | | | **Emotional HRQOL** | | | **School HRQOL** | | |  |  |  |  |  |  |  |  |  |  |  |  |
| --- | --- | --- | --- | --- | --- | --- | --- | --- | --- | --- | --- | --- | --- | --- | --- | --- | --- | --- | --- | --- | --- |
|  | **β** | **95% CI** | ***p*** | **β** | **95% CI** | ***p*** | **β** | **95% CI** | ***p*** |  |  |  |  |  |  |  |  |  |  |  |  |
| **Unadjusted models** |  |  |  |  |  |  |  |  |  |  |  |  |  |  |  |  |  |  |  |  |  |
| Active transport (+30 min/day) [a] | 0.25 | –0.37, 0.88 | 0.423 | 0.17 | –0.49, 0.83 | 0.617 | 0.30 | –0.39, 0.98 | 0.396 |  |  |  |  |  |  |  |  |  |  |  |  |
| **Adjusted models** |  |  |  |  |  |  |  |  |  |  |  |  |  |  |  |  |  |  |  |  |  |
| Active transport (+30 min/day) [a] | 0.07 | –0.44, 0.59 | 0.779 | 0.08 | –0.41, 0.57 | 0.757 | 0.15 | –0.52, 0.83 | 0.658 |  |  |  |  |  |  |  |  |  |  |  |  |
| Age (months) | –0.09 | –0.22, 0.05 | 0.199 | 0.10 | –0.03, 0.23 | 0.116 | 0.04 | –0.10, 0.18 | 0.604 |  |  |  |  |  |  |  |  |  |  |  |  |
| Sex (female) | 0.14 | –0.96, 1.25 | 0.799 | –2.04 | –3.15, –0.93 | <0.001 | 0.79 | –0.38, 1.95 | 0.186 |  |  |  |  |  |  |  |  |  |  |  |  |
| BMI (z–score) | –0.60 | –1.04, –0.16 | 0.007 | 0.44 | –0.08, 0.96 | 0.099 | –0.33 | –0.72, 0.07 | 0.102 |  |  |  |  |  |  |  |  |  |  |  |  |
| Pubertal development [b] | –0.27 | –1.44, 0.90 | 0.652 | –0.82 | –2.02, 0.38 | 0.181 | –1.28 | –2.57, 0.00 | 0.050 |  |  |  |  |  |  |  |  |  |  |  |  |
| Socioeconomic position (z–score) | 1.06 | 0.55, 1.56 | <0.001 | 0.46 | –0.05, 0.97 | 0.080 | 1.05 | 0.44, 1.65 | 0.001 |  |  |  |  |  |  |  |  |  |  |  |  |
| School attendance on day of TUD (No) | 0.65 | –0.34, 1.63 | 0.197 | –0.34 | –1.32, 0.63 | 0.492 | –0.14 | –1.21, 0.92 | 0.796 |  |  |  |  |  |  |  |  |  |  |  |  |
| Season of measurement [c] | –0.05 | –0.47, 0.36 | 0.797 | –0.09 | –0.51, 0.33 | 0.679 | –0.30 | –0.75, 0.15 | 0.190 |  |  |  |  |  |  |  |  |  |  |  |  |
| PedsQL outcome at 10–11y [d] | 0.61 | 0.57, 0.65 | <0.001 | 0.61 | 0.58, 0.65 | <0.001 | 0.42 | 0.38, 0.47 | <0.001 |  |  |  |  |  |  |  |  |  |  |  |  |
| PA = physical activity; HRQOL = health related quality of life, measured using PedsQL scales; β = model coefficient; CI = confidence interval. | | | | | | | | | | | | | | | | |  |  |  |  |  |
| a. The coefficients presented in this table for this domain of PA represent the predicted effect associated with an increase of 30 min/day. | | | | | | | | | | | | | | | | | | | | | |
| b. The pubertal development scale ranged from 1 (least developed) to 4 (most developed). | | | | | | | | | | |  |  |  |  |  |  |  |  |  |  |  |
| c. Season of TUD completion was categorized as: 1= Winter; 2=Spring; 3=Summer; 4=Autumn. | | | | | | | | | | | |  |  |  |  |  |  |  |  |  |  |
| d. This variable refers to the corresponding PedsQL domain measured at 10–11y (e.g. the PedsQL physical scale at 10–11y was included in the physical HRQOL model, and so forth). | | | | | | | | | | | | | | | |  |  |  |  |  |  |

**Table A8A: Associations between the absolute duration of active chores/work at 10–11y and main HRQOL outcomes at 12–13y**

|  | **Physical HRQOL** | | | **Psychosocial HRQOL [a]** | | | **Total HRQOL [b]** | | |  |  |  |  |  |  |  |  |  |  |  |  |
| --- | --- | --- | --- | --- | --- | --- | --- | --- | --- | --- | --- | --- | --- | --- | --- | --- | --- | --- | --- | --- | --- |
|  | **β** | **95% CI** | ***p*** | **β** | **95% CI** | ***p*** | **β** | **95% CI** | ***p*** |  |  |  |  |  |  |  |  |  |  |  |  |
| **Unadjusted models** |  |  |  |  |  |  |  |  |  |  |  |  |  |  |  |  |  |  |  |  |  |
| Active chores/work (+30 min/day) [c] | –0.06 | –0.39, 0.27 | 0.717 | –0.04 | –0.47, 0.39 | 0.864 | –0.02 | –0.38, 0.34 | 0.909 |  |  |  |  |  |  |  |  |  |  |  |  |
| **Adjusted models** |  |  |  |  |  |  |  |  |  |  |  |  |  |  |  |  |  |  |  |  |  |
| Active chores/work (+30 min/day) [c] | 0.02 | –0.31, 0.36 | 0.889 | 0.05 | –0.28, 0.39 | 0.757 | 0.12 | –0.18, 0.42 | 0.420 |  |  |  |  |  |  |  |  |  |  |  |  |
| Age (months) | 0.00 | –0.10, 0.09 | 0.977 | 0.00 | –0.11, 0.11 | 0.975 | 0.00 | –0.10, 0.09 | 0.926 |  |  |  |  |  |  |  |  |  |  |  |  |
| Sex (female) | –0.19 | –0.98, 0.60 | 0.635 | –0.84 | –1.80, 0.11 | 0.082 | –0.43 | –1.22, 0.35 | 0.280 |  |  |  |  |  |  |  |  |  |  |  |  |
| BMI (z–score) | –0.13 | –0.45, 0.19 | 0.424 | –0.08 | –0.52, 0.36 | 0.708 | –0.03 | –0.35, 0.29 | 0.856 |  |  |  |  |  |  |  |  |  |  |  |  |
| Pubertal development [d] | –0.76 | –1.64, 0.13 | 0.096 | –0.67 | –1.70, 0.35 | 0.199 | –0.94 | –1.79, –0.09 | 0.030 |  |  |  |  |  |  |  |  |  |  |  |  |
| Socioeconomic position (z–score) | 0.69 | 0.33, 1.05 | <0.001 | 0.65 | 0.23, 1.07 | 0.003 | 0.75 | 0.39, 1.10 | <0.001 |  |  |  |  |  |  |  |  |  |  |  |  |
| School attendance on day of TUD (No) | 0.23 | –0.48, 0.94 | 0.522 | 0.15 | –0.70, 1.01 | 0.725 | 0.27 | –0.42, 0.97 | 0.439 |  |  |  |  |  |  |  |  |  |  |  |  |
| Season of measurement [e] | 0.07 | –0.22, 0.36 | 0.643 | –0.05 | –0.40, 0.31 | 0.804 | –0.01 | –0.30, 0.28 | 0.932 |  |  |  |  |  |  |  |  |  |  |  |  |
| PedsQL outcome at 10–11y [f] | 0.64 | 0.58, 0.70 | <0.001 | 0.67 | 0.64, 0.70 | <0.001 | 0.71 | 0.67, 0.75 | <0.001 |  |  |  |  |  |  |  |  |  |  |  |  |
| PA = physical activity; HRQOL = health related quality of life, measured using PedsQL scales; β = model coefficient; CI = confidence interval. | | | | | | | | | | | | | | | | |  |  |  |  |  |
| a. The psychosocial HRQOL summary scale is a composite of social, emotional and school HRQOL | | | | | | | | | | | |  |  |  |  |  |  |  |  |  |  |
| b. The total HRQOL summary scale is a composite of physical, social, emotional and school HRQOL | | | | | | | | | | | |  |  |  |  |  |  |  |  |  |  |
| c. The coefficients presented in this table for this domain of PA represent the predicted effect associated with an increase of 30 min/day. | | | | | | | | | | | | | | | | | | | | | |
| d. The pubertal development scale ranged from 1 (least developed) to 4 (most developed). | | | | | | | | | | |  |  |  |  |  |  |  |  |  |  |  |
| e. Season of TUD completion was categorized as: 1= Winter; 2=Spring; 3=Summer; 4=Autumn. | | | | | | | | | | | |  |  |  |  |  |  |  |  |  |  |
| f. This variable refers to the corresponding PedsQL domain measured at 10-11y (e.g. the PedsQL physical scale at 10-11y was included in the physical HRQOL model, and so forth). | | | | | | | | | | | | | | | |  |  |  |  |  |  |

**Table A8B: Associations between the absolute duration of active chores/work at 10–11y and additional HRQOL outcomes at 12–13y**

|  | **Social HRQOL** | | | **Emotional HRQOL** | | | **School HRQOL** | | |  |  |  |  |  |  |  |  |  |  |  |  |
| --- | --- | --- | --- | --- | --- | --- | --- | --- | --- | --- | --- | --- | --- | --- | --- | --- | --- | --- | --- | --- | --- |
|  | **β** | **95% CI** | ***p*** | **β** | **95% CI** | ***p*** | **β** | **95% CI** | ***p*** |  |  |  |  |  |  |  |  |  |  |  |  |
| **Unadjusted models** |  |  |  |  |  |  |  |  |  |  |  |  |  |  |  |  |  |  |  |  |  |
| Active chores/work (+30 min/day) [a] | –0.18 | –0.67, 0.30 | 0.460 | 0.08 | –0.38, 0.55 | 0.725 | –0.02 | –0.45, 0.41 | 0.922 |  |  |  |  |  |  |  |  |  |  |  |  |
| **Adjusted models** |  |  |  |  |  |  |  |  |  |  |  |  |  |  |  |  |  |  |  |  |  |
| Active chores/work (+30 min/day) [a] | –0.16 | –0.55, 0.23 | 0.426 | 0.26 | –0.13, 0.65 | 0.192 | 0.04 | –0.36, 0.44 | 0.851 |  |  |  |  |  |  |  |  |  |  |  |  |
| Age (months) | –0.08 | –0.22, 0.05 | 0.210 | 0.10 | –0.03, 0.23 | 0.120 | 0.04 | –0.10, 0.18 | 0.595 |  |  |  |  |  |  |  |  |  |  |  |  |
| Sex (female) | 0.17 | –0.93, 1.27 | 0.764 | –2.09 | –3.21, –0.98 | <0.001 | 0.78 | –0.40, 1.95 | 0.194 |  |  |  |  |  |  |  |  |  |  |  |  |
| BMI (z–score) | –0.60 | –1.04, –0.16 | 0.007 | 0.44 | –0.08, 0.95 | 0.099 | –0.33 | –0.72, 0.07 | 0.104 |  |  |  |  |  |  |  |  |  |  |  |  |
| Pubertal development [b] | –0.28 | –1.45, 0.90 | 0.644 | –0.80 | –2.00, 0.40 | 0.192 | –1.29 | –2.57, –0.01 | 0.049 |  |  |  |  |  |  |  |  |  |  |  |  |
| Socioeconomic position (z–score) | 1.05 | 0.55, 1.56 | <0.001 | 0.47 | –0.04, 0.98 | 0.073 | 1.05 | 0.44, 1.65 | 0.001 |  |  |  |  |  |  |  |  |  |  |  |  |
| School attendance on day of TUD (No) | 0.72 | –0.28, 1.72 | 0.157 | –0.48 | –1.47, 0.51 | 0.344 | –0.18 | –1.26, 0.91 | 0.751 |  |  |  |  |  |  |  |  |  |  |  |  |
| Season of measurement [c] | –0.05 | –0.47, 0.36 | 0.798 | –0.09 | –0.51, 0.33 | 0.674 | –0.30 | –0.75, 0.15 | 0.189 |  |  |  |  |  |  |  |  |  |  |  |  |
| PedsQL outcome at 10–11y [d] | 0.61 | 0.57, 0.65 | <0.001 | 0.61 | 0.58, 0.65 | <0.001 | 0.42 | 0.38, 0.47 | <0.001 |  |  |  |  |  |  |  |  |  |  |  |  |
| PA = physical activity; HRQOL = health related quality of life, measured using PedsQL scales; β = model coefficient; CI = confidence interval. | | | | | | | | | | | | | | | | |  |  |  |  |  |
| a. The coefficients presented in this table for this domain of PA represent the predicted effect associated with an increase of 30 min/day. | | | | | | | | | | | | | | | | | | | | | |
| b. The pubertal development scale ranged from 1 (least developed) to 4 (most developed). | | | | | | | | | | |  |  |  |  |  |  |  |  |  |  |  |
| c. Season of TUD completion was categorized as: 1= Winter; 2=Spring; 3=Summer; 4=Autumn. | | | | | | | | | | | |  |  |  |  |  |  |  |  |  |  |
| d. This variable refers to the corresponding PedsQL domain measured at 10-11y (e.g. the PedsQL physical scale at 10-11y was included in the physical HRQOL model, and so forth). | | | | | | | | | | | | | | | |  |  |  |  |  |  |
