# Supplementary material for: Cross-sectional and longitudinal associations of domain-specific physical activity composition with health-related quality of life in childhood and adolescence in Australia
Source: Int J Behav Nutr Phys Act. 2023 Jun 6;20:67. doi: 10.1186/s12966-023-01466-6 (PMC10242981; doi:10.1186/s12966-023-01466-6)
Supplement: Supplementary file 2 — Additional file 2. Full results of compositional models. Provides the full results of all compositional models, including subcomponents of psychosocial health-related quality of life, unadjusted estimates and covariates. [file 12966_2023_1466_MOESM2_ESM.docx]

**Cross-sectional and longitudinal associations of domain-specific physical activity composition with health-related quality of life in childhood and adolescence in Australia**

**Additional file 2:** Full results of compositional models

***Cross-sectional models***

**Table B1: Associations between the overall composition of PA at 10-11y and HRQOL outcomes at 10-11y, controlling for covariates, LSAC data**

|  | **Physical HRQOL** | | | **Social HRQOL** | | | **Emotional HRQOL** | | | **School HRQOL** | | | **Psychosocial HRQOL[a]** | | | **Total HRQOL [b]** | | |  |  |
| --- | --- | --- | --- | --- | --- | --- | --- | --- | --- | --- | --- | --- | --- | --- | --- | --- | --- | --- | --- | --- |
|  | **df** | **χ2** | **p-value** | **df** | **χ2** | **p-value** | **df** | **χ2** | **p-value** | **df** | **χ2** | **p-value** | **df** | **χ2** | **p-value** | **df** | **χ2** | **p-value** |  |  |
| **Overall PA composition [c]** | 3 | 9.01 | 0.029* | 3 | 11.3 | 0.010* | 3 | 7.89 | 0.048* | 3 | 2.55 | 0.467 | 3 | 11.37 | 0.010* | 3 | 11.29 | 0.010* |  |  |
| PA = physical activity; HRQOL = health related quality of life, measured using PedsQL scales; LSAC = Longitudinal Study of Australian Children; df = degrees of freedom; χ2 = chi square statistic | | | | | | | | | | | | | | | | | | | | |
| a. The psychosocial HRQOL summary scale is a composite of social, emotional and school HRQOL | | | | | | | | | | | | | | | | | | | | |
| b. The total HRQOL summary scale is a composite of physical, social, emotional and school HRQOL | | | | | | | | | | | | | | | | | | | | |
| c. This row contains output from the ANOVA table of model fit which was used to determine whether the collective set of isometic log-ratios (*ilrs)* had a statistically significant association with HRQOL outcomes | | | | | | | | | | | | | | | | | | | | |

**Table B2: Associations between +30 minutes spent in each domain of PA (relative to remaining domains) at 10-11y and HRQOL outcomes at 10-11y, not adjusted for covariates, LSAC data**

|  | **Physical HRQOL** | | | | **Social HRQOL** | | | | **Emotional HRQOL** | | | | **School HRQOL** | | | | **Psychosocial HRQOL [a]** | | | | **Total HRQOL [b]** | | | | |
| --- | --- | --- | --- | --- | --- | --- | --- | --- | --- | --- | --- | --- | --- | --- | --- | --- | --- | --- | --- | --- | --- | --- | --- | --- | --- |
|  | **Estimate** | | **95% CI** | | **Estimate** | | **95% CI** | | **Estimate** | | **95% CI** | | **Estimate** | | **95% CI** | | **Estimate** | | **95% CI** | | **Estimate** | | **95% CI** | | |
| Non-organized PA [c] | 0.13 | -0.33 | | 0.59 | 0.14 | -0.38 | | 0.66 | 0.05 | -0.46 | | 0.56 | -0.06 | -0.56 | | 0.44 | 0.11 | -0.34 | | 0.56 | 0.14 | -0.26 | | 0.53 | |
| Organized PA [c] | 0.36 | 0.05 | | 0.67 | 0.60 | 0.25 | | 0.96 | 0.47 | 0.13 | | 0.81 | 0.32 | -0.01 | | 0.66 | 0.51 | 0.21 | | 0.82 | 0.47 | 0.21 | | 0.74 | |
| Active transport [c] | -0.30 | -0.76 | | 0.16 | -0.19 | -0.72 | | 0.33 | 0.03 | -0.49 | | 0.54 | -0.02 | -0.52 | | 0.49 | -0.05 | -0.50 | | 0.41 | -0.23 | -0.63 | | 0.17 | |
| Active chores/work [c] | -0.22 | -0.59 | | 0.16 | -0.59 | -1.02 | | -0.17 | -0.58 | -0.99 | | -0.17 | -0.30 | -0.71 | | 0.11 | -0.61 | -0.97 | | -0.24 | -0.42 | -0.74 | | -0.09 | |
| PA = physical activity; HRQOL = health related quality of life, measured using PedsQL scales; LSAC = Longitudinal Study of Australian Children; CI = confidence interval. | | | | | | | | | | | | | | | | | | | | | | | | |  |
| a. The psychosocial HRQOL summary scale is a composite of social, emotional and school HRQOL | | | | | | | | | | | | | | | | | | | | | | | | |  |
| b. The total HRQOL summary scale is a composite of physical, social, emotional and school HRQOL | | | | | | | | | | | | | | | | | | | | | | | | |  |
| c. The coefficients presented in these rows represent associations between each individual PA domain and HRQOL outcomes, relative to all other domains (multiplicative total was included in the model).  **Table B3A: Associations between compositional isometric log ratios at 10-11y and main HRQOL outcomes at 10-11y, including covariates, LSAC data**   \|  \| **Physical HRQOL** \| \| \| \| \| \| \| **Psychosocial HRQOL [a]** \| \| \| \| \| \| **Psychosocial HRQOL [b]** \| \| \| \| \| \| \| \| \| \| --- \| --- \| --- \| --- \| --- \| --- \| --- \| --- \| --- \| --- \| --- \| --- \| --- \| --- \| --- \| --- \| --- \| --- \| --- \| --- \| --- \| --- \| --- \| \|  \| **β** \| \| **95% CI** \| \| \| ***p*** \| \| **95% CI** \| \| **95% CI** \| \| ***p*** \| \| **β** \| \| **95% CI** \| \| \| \| ***p*** \| \| \| \| **Composition isometric log ratio** \| \| \| \| \|  \| \| \| \| \| \| \| \| \| \| \| \|  \| \| \| \| \| \| \| Non-organized PA vs remaining \| 0.05 \| -0.13 \| \| 0.24 \| \| \| 0.577 \| 0.05 \| -0.19 \| \| 0.29 \| \| 0.675 \| 0.05 \| -0.15 \| \| \| \| 0.26 \| \| \| 0.606 \| \| Organized PA vs Active transport & Active work/chores \| 0.29 \| 0.11 \| \| 0.48 \| \| \| 0.002 \| 0.37 \| 0.12 \| \| 0.62 \| \| 0.004 \| 0.35 \| 0.14 \| \| \| \| 0.56 \| \| \| 0.001 \| \| Active transport vs Active work/chores \| -0.09 \| -0.3 \| \| 0.12 \| \| \| 0.408 \| 0.23 \| -0.07 \| \| 0.52 \| \| 0.130 \| 0.07 \| -0.17 \| \| \| \| 0.31 \| \| \| 0.579 \| \| **Covariates** \| \| \| \| \| \| \| \| \| \| \| \| \| \| \| \| \| \|  \| \| \|  \| \| \| Age (months) \| 0.02 \| -0.10 \| \| 0.14 \| \| \| 0.767 \| 0.12 \| -0.03 \| \| 0.27 \| \| 0.128 \| 0.08 \| -0.05 \| \| \| \| 0.21 \| \| \| 0.238 \| \| Sex (female) \| -0.23 \| -1.12 \| \| 0.67 \| \| \| 0.620 \| -0.41 \| -1.62 \| \| 0.79 \| \| 0.503 \| -0.43 \| -1.46 \| \| \| \| 0.59 \| \| \| 0.408 \| \| BMI (z-score) \| -0.75 \| -1.11 \| \| -0.40 \| \| \| <0.001 \| -0.28 \| -0.73 \| \| 0.17 \| \| 0.229 \| -0.63 \| -1.05 \| \| \| \| -0.21 \| \| \| 0.003 \| \| Pubertal development [c] \| -1.49 \| -2.51 \| \| -0.48 \| \| \| <0.001 \| -1.97 \| -3.31 \| \| -0.63 \| \| 0.004 \| -1.83 \| -2.96 \| \| \| \| -0.69 \| \| \| 0.002 \| \| Socioeconomic position (z-score) \| 0.25 \| -0.14 \| \| 0.63 \| \| \| 0.215 \| 0.70 \| 0.16 \| \| 1.25 \| \| 0.012 \| 0.69 \| 0.24 \| \| \| \| 1.13 \| \| \| 0.003 \| \| School attendance on day of TUD (No) \| -0.74 \| -1.55 \| \| 0.08 \| \| \| 0.077 \| -0.77 \| -1.86 \| \| 0.33 \| \| 0.170 \| -0.73 \| -1.65 \| \| \| \| 0.20 \| \| \| 0.123 \| \| Season of measurement \| \| \| \| \| \| \| \| \| \| \| \| \| \| \| \| \| \| \| \| \| \| \| \| Spring \| 0.54 \| -0.44 \| \| 1.51 \| \| \| 0.278 \| -0.82 \| -2.16 \| \| 0.51 \| \| 0.226 \| -0.37 \| -1.49 \| \| \| \| 0.76 \| \| \| 0.521 \| \| Summer \| 0.4 \| -2.76 \| \| 3.57 \| \| \| 0.802 \| 0.09 \| -3.38 \| \| 3.55 \| \| 0.961 \| -0.49 \| -3.67 \| \| \| \| 2.69 \| \| \| 0.763 \| \| Autumn \| 0.71 \| -0.33 \| \| 1.74 \| \| \| 0.180 \| 0.70 \| -0.66 \| \| 2.05 \| \| 0.314 \| 0.65 \| -0.48 \| \| \| \| 1.78 \| \| \| 0.259 \| \| Multiplicative total \| 0.43 \| 0.22 \| \| 0.64 \| \| \| <0.001 \| 0.55 \| 0.28 \| \| 0.83 \| \| <0.001 \| 0.56 \| 0.32 \| \| \| \| 0.79 \| \| \| <0.001 \| | | | | | | | | | | | | | | | | | | | | | | | | |  |

PA = physical activity; HRQOL = health related quality of life, measured using PedsQL scales; LSAC = Longitudinal Study of Australian Children; β = model coefficient; CI = confidence interval.

1. The psychosocial HRQOL summary scale is a composite of social, emotional and school HRQOL
2. The total HRQOL summary scale is a composite of physical, social, emotional and school HRQOL
3. The pubertal development scale ranged from 1 (least developed) to 4 (most developed)

| **Table B3B: Associations between compositional isometric log ratios at 10-11y and additional HRQOL outcomes at 10-11y, including covariates, LSAC data**   \|  \| **Social HRQOL** \| \| \| \| \| \| \| **Emotional HRQOL** \| \| \| \| \| \| **School HRQOL** \| \| \| \| \| \| \| \| \| \| --- \| --- \| --- \| --- \| --- \| --- \| --- \| --- \| --- \| --- \| --- \| --- \| --- \| --- \| --- \| --- \| --- \| --- \| --- \| --- \| --- \| --- \| --- \| \|  \| **β** \| \| **95% CI** \| \| \| ***p*** \| \| **β** \| \| **95% CI** \| \| ***p*** \| \| **β** \| \| **95% CI** \| \| \| \| ***p*** \| \| \| \| **Composition isometric log ratio** \| \| \| \| \|  \| \| \| \| \| \| \| \| \| \| \| \|  \| \| \| \| \| \| \| Non-organized PA vs remaining \| 0.04 \| -0.23 \| \| 0.32 \| \| \| 0.761 \| 0.03 \| -0.24 \| \| 0.31 \| \| 0.815 \| 0.03 \| -0.21 \| \| \| \| 0.27 \| \| \| 0.793 \| \| Organized PA vs Active transport & Active work/chores \| 0.45 \| 0.17 \| \| 0.73 \| \| \| 0.002 \| 0.35 \| 0.05 \| \| 0.64 \| \| 0.021 \| 0.15 \| -0.09 \| \| \| \| 0.39 \| \| \| 0.219 \| \| Active transport vs Active work/chores \| 0.17 \| -0.17 \| \| 0.50 \| \| \| 0.323 \| 0.25 \| -0.09 \| \| 0.59 \| \| 0.156 \| 0.13 \| -0.15 \| \| \| \| 0.41 \| \| \| 0.371 \| \| **Covariates** \| \| \| \| \| \| \| \| \| \| \| \| \| \| \| \| \| \|  \| \| \|  \| \| \| Age (months) \| 0.11 \| -0.06 \| \| 0.29 \| \| \| 0.201 \| 0.10 \| -0.08 \| \| 0.27 \| \| 0.287 \| -0.02 \| -0.17 \| \| \| \| 0.13 \| \| \| 0.821 \| \| Sex (female) \| -0.32 \| -1.66 \| \| 1.03 \| \| \| 0.646 \| -0.28 \| -1.65 \| \| 1.09 \| \| 0.687 \| 1.54 \| 0.38 \| \| \| \| 2.69 \| \| \| 0.009 \| \| BMI (z-score) \| -0.57 \| -1.23 \| \| 0.09 \| \| \| 0.088 \| -0.03 \| -0.49 \| \| 0.44 \| \| 0.907 \| -0.28 \| -0.71 \| \| \| \| 0.15 \| \| \| 0.201 \| \| Pubertal development [a] \| -2.37 \| -3.88 \| \| -0.87 \| \| \| 0.002 \| -1.56 \| -3.07 \| \| -0.06 \| \| 0.042 \| -1.45 \| -2.71 \| \| \| \| -0.18 \| \| \| 0.025 \| \| Socioeconomic position (z-score) \| 1.19 \| 0.57 \| \| 1.81 \| \| \| <0.001 \| 0.12 \| -0.49 \| \| 0.73 \| \| 0.697 \| 1.57 \| 1.02 \| \| \| \| 2.12 \| \| \| <0.001 \| \| School attendance on day of TUD (No) \| -0.63 \| -1.87 \| \| 0.62 \| \| \| 0.324 \| -0.97 \| -2.22 \| \| 0.28 \| \| 0.128 \| -0.72 \| -1.79 \| \| \| \| 0.36 \| \| \| 0.191 \| \| Season of measurement \| \| \| \| \| \| \| \| \| \| \| \| \| \| \| \| \| \| \| \| \| \| \| \| Spring \| -0.68 \| -2.19 \| \| 0.82 \| \| \| 0.374 \| -0.73 \| -2.26 \| \| 0.80 \| \| 0.351 \| -0.59 \| -1.96 \| \| \| \| 0.78 \| \| \| 0.399 \| \| Summer \| 1.13 \| -2.52 \| \| 4.77 \| \| \| 0.545 \| -1.09 \| -5.65 \| \| 3.47 \| \| 0.641 \| -1.87 \| -6.25 \| \| \| \| 2.52 \| \| \| 0.404 \| \| Autumn \| 1.30 \| -0.24 \| \| 2.84 \| \| \| 0.099 \| -0.07 \| -1.63 \| \| 1.50 \| \| 0.935 \| 3.25 \| 2.03 \| \| \| \| 4.46 \| \| \| <0.001 \| \| Multiplicative total \| 0.64 \| 0.33 \| \| 0.95 \| \| \| <0.001 \| 0.45 \| 0.13 \| \| 0.76 \| \| 0.006 \| 0.67 \| 0.41 \| \| \| \| 0.94 \| \| \| <0.001 \| |
| --- | --- | --- | --- | --- | --- | --- | --- | --- | --- | --- | --- | --- | --- | --- | --- | --- | --- | --- | --- | --- | --- | --- | --- | --- | --- | --- | --- | --- | --- | --- | --- | --- | --- | --- | --- | --- | --- | --- | --- | --- | --- | --- | --- | --- | --- | --- | --- | --- | --- | --- | --- | --- | --- | --- | --- | --- | --- | --- | --- | --- | --- | --- | --- | --- | --- | --- | --- | --- | --- | --- | --- | --- | --- | --- | --- | --- | --- | --- | --- | --- | --- | --- | --- | --- | --- | --- | --- | --- | --- | --- | --- | --- | --- | --- | --- | --- | --- | --- | --- | --- | --- | --- | --- | --- | --- | --- | --- | --- | --- | --- | --- | --- | --- | --- | --- | --- | --- | --- | --- | --- | --- | --- | --- | --- | --- | --- | --- | --- | --- | --- | --- | --- | --- | --- | --- | --- | --- | --- | --- | --- | --- | --- | --- | --- | --- | --- | --- | --- | --- | --- | --- | --- | --- | --- | --- | --- | --- | --- | --- | --- | --- | --- | --- | --- | --- | --- | --- | --- | --- | --- | --- | --- | --- | --- | --- | --- | --- | --- | --- | --- | --- | --- | --- | --- | --- | --- | --- | --- | --- | --- | --- | --- | --- | --- | --- | --- | --- | --- | --- | --- | --- | --- | --- | --- | --- | --- | --- | --- | --- | --- | --- | --- | --- | --- | --- | --- | --- | --- | --- | --- | --- | --- | --- | --- | --- | --- | --- | --- | --- | --- | --- | --- | --- | --- | --- | --- | --- | --- | --- | --- | --- | --- | --- | --- | --- | --- | --- | --- | --- | --- | --- | --- | --- | --- | --- | --- | --- | --- | --- | --- | --- | --- | --- | --- | --- | --- | --- | --- | --- | --- | --- | --- | --- | --- | --- | --- | --- | --- | --- | --- | --- | --- | --- | --- | --- | --- | --- | --- | --- | --- | --- | --- | --- | --- | --- | --- | --- | --- | --- | --- | --- | --- | --- | --- | --- | --- | --- | --- | --- | --- | --- | --- | --- | --- | --- | --- | --- | --- | --- | --- | --- | --- | --- | --- | --- | --- | --- | --- | --- | --- | --- | --- | --- | --- | --- | --- | --- | --- | --- | --- | --- | --- | --- | --- | --- | --- | --- | --- | --- | --- | --- | --- | --- | --- | --- | --- | --- | --- | --- | --- | --- | --- | --- | --- | --- | --- | --- | --- | --- | --- | --- | --- | --- | --- | --- | --- | --- | --- | --- | --- | --- | --- | --- | --- | --- | --- | --- | --- | --- | --- | --- | --- | --- | --- | --- | --- | --- | --- | --- | --- | --- | --- | --- | --- | --- | --- | --- | --- | --- | --- | --- | --- | --- | --- |

PA = physical activity; HRQOL = health related quality of life, measured using PedsQL scales; LSAC = Longitudinal Study of Australian Children; β = model coefficient; CI = confidence interval.

1. The pubertal development scale ranged from 1 (least developed) to 4 (most developed)

***Longitudinal models***

**Table B4: Associations between the overall composition of PA at 10-11y and HRQOL outcomes at 12-13y, controlling for covariates, LSAC data**

|  | **Physical HRQOL** | | | **Social HRQOL** | | | **Emotional HRQOL** | | | **School HRQOL** | | | **Psychosocial HRQOL [a]** | | | **Total HRQOL [b]** | | |  |  |
| --- | --- | --- | --- | --- | --- | --- | --- | --- | --- | --- | --- | --- | --- | --- | --- | --- | --- | --- | --- | --- |
|  | **df** | **χ2** | **p-value** | **df** | **χ2** | **p-value** | **df** | **χ2** | **p-value** | **df** | **χ2** | **p-value** | **df** | **χ2** | **p-value** | **df** | **χ2** | **p-value** |  |  |
| **Overall PA composition [c]** | 3 | 5.43 | 0.143 | 3 | 1.59 | 0.662 | 3 | 1.35 | 0.719 | 3 | 1.31 | 0.727 | 3 | 1.38 | 0.71 | 3 | 2.35 | 0.504 |  |  |
| PA = physical activity; HRQOL = health related quality of life, measured using PedsQL scales; LSAC = Longitudinal Study of Australian Children; df = degrees of freedom; χ2 = chi square statistic | | | | | | | | | | | | | | | | | | | | |
| a. The psychosocial HRQOL summary scale is a composite of social, emotional and school HRQOL | | | | | | | | | | | | | | | | | | | | |
| b. The total HRQOL summary scale is a composite of physical, social, emotional and school HRQOL | | | | | | | | | | | | | | | | | | | | |
| c. This row contains output from the ANOVA table of model fit which was used to determine whether the collective set of isometic log-ratios (*ilrs)* had a statistically significant association with HRQOL outcomes | | | | | | | | | | | | | | | | | | | | |

**Table B5: Associations between +30 minutes spent in each domain of PA (relative to remaining domains) at 10-11y and HRQOL outcomes at 12-13y, not adjusted**

|  | **Physical HRQOL** | | | | **Social HRQOL** | | | | **Emotional HRQOL** | | | | **School HRQOL** | | | | **Psychosocial HRQOL [a]** | | | | **Total HRQOL [b]** | | | |
| --- | --- | --- | --- | --- | --- | --- | --- | --- | --- | --- | --- | --- | --- | --- | --- | --- | --- | --- | --- | --- | --- | --- | --- | --- |
|  | **Estimate** | | **95% CI** | | **Estimate** | | **95% CI** | | **Estimate** | | **95% CI** | | **Estimate** | | **95% CI** | | **Estimate** | | **95% CI** | | **Estimate** | | **95% CI** | |
| Non-organized PA [c] | 0.22 | -0.28 | | 0.72 | 0.39 | -0.07 | | 0.86 | 0.29 | -0.13 | | 0.72 | 0.46 | -0.09 | | 1.00 | 0.27 | -0.26 | | 0.80 | -0.34 | -0.88 | | 0.20 |
| Organized PA [c] | 0.39 | 0.05 | | 0.73 | 0.40 | 0.08 | | 0.72 | 0.42 | 0.13 | | 0.71 | 0.33 | -0.04 | | 0.69 | 0.44 | 0.07 | | 0.80 | 0.20 | -0.17 | | 0.57 |
| Active transport [c] | -0.49 | -1.00 | | 0.02 | -0.28 | -0.76 | | 0.19 | -0.43 | -0.86 | | -0.01 | -0.13 | -0.68 | | 0.42 | -0.33 | -0.86 | | 0.21 | 0.13 | -0.42 | | 0.68 |
| Active chores/work [c] | -0.14 | -0.55 | | 0.27 | -0.48 | -0.87 | | -0.09 | -0.28 | -0.63 | | 0.06 | -0.58 | -1.03 | | -0.14 | -0.38 | -0.82 | | 0.05 | -0.09 | -0.53 | | 0.36 |

PA = physical activity; HRQOL = health related quality of life, measured using PedsQL scales; LSAC = Longitudinal Study of Australian Children; CI = confidence interval.

1. The psychosocial HRQOL summary scale is a composite of social, emotional and school HRQOL
2. The total HRQOL summary scale is a composite of physical, social, emotional and school HRQOL
3. The coefficients presented in these rows represent associations between each individual PA domain and HRQOL outcomes, relative to all other domains (multiplicative total was included in the model).

| **Table B6A: Associations between compositional isometric log ratios at 10-11y and main HRQOL outcomes at 12-13y, including covariates, LSAC data**   \|  \| **Physical HRQOL** \| \| \| \| \| \| \| **Psychosocial HRQOL [a]** \| \| \| \| \| \| **Total HRQOL [b]** \| \| \| \| \| \| \| \| \| \| --- \| --- \| --- \| --- \| --- \| --- \| --- \| --- \| --- \| --- \| --- \| --- \| --- \| --- \| --- \| --- \| --- \| --- \| --- \| --- \| --- \| --- \| --- \| \|  \| **β** \| \| **95% CI** \| \| \| ***p*** \| \| **95% CI** \| \| **95% CI** \| \| ***p*** \| \| **β** \| \| **95% CI** \| \| \| \| ***p*** \| \| \| \| **Composition isometric log ratio** \| \| \| \| \|  \| \| \| \| \| \| \| \| \| \| \| \|  \| \| \| \| \| \| \| Non-organized PA vs remaining \| -0.01 \| -0.17 \| \| 0.15 \| \| \| 0.877 \| 0.09 \| -0.10 \| \| 0.28 \| \| 0.339 \| 0.04 \| -0.12 \| \| \| \| 0.19 \| \| \| 0.637 \| \| Organized PA vs Active transport & Active work/chores \| 0.15 \| -0.02 \| \| 0.31 \| \| \| 0.081 \| 0.08 \| -0.12 \| \| 0.28 \| \| 0.419 \| 0.07 \| -0.10 \| \| \| \| 0.23 \| \| \| 0.421 \| \| Active transport vs Active work/chores \| -0.15 \| -0.35 \| \| 0.04 \| \| \| 0.126 \| -0.04 \| -0.27 \| \| 0.20 \| \| 0.769 \| -0.13 \| -0.32 \| \| \| \| 0.06 \| \| \| 0.183 \| \| **Covariates** \| \| \| \| \| \| \| \| \| \| \| \| \| \| \| \| \| \|  \| \| \|  \| \| \| Age (months) \| 0.01 \| -0.09 \| \| 0.12 \| \| \| 0.813 \| 0.03 \| -0.09 \| \| 0.15 \| \| 0.622 \| 0.02 \| -0.09 \| \| \| \| 0.12 \| \| \| 0.768 \| \| Sex (female) \| -0.19 \| -0.99 \| \| 0.61 \| \| \| 0.642 \| -0.76 \| -1.71 \| \| 0.20 \| \| 0.122 \| -0.40 \| -1.19 \| \| \| \| 0.39 \| \| \| 0.325 \| \| BMI (z-score) \| -0.12 \| -0.44 \| \| 0.21 \| \| \| 0.483 \| -0.06 \| -0.51 \| \| 0.39 \| \| 0.805 \| -0.01 \| -0.34 \| \| \| \| 0.31 \| \| \| 0.934 \| \| Pubertal development [c] \| -0.81 \| -1.69 \| \| 0.08 \| \| \| 0.076 \| -0.65 \| -1.67 \| \| 0.38 \| \| 0.215 \| -0.95 \| -1.79 \| \| \| \| -0.10 \| \| \| 0.028 \| \| Socioeconomic position (z-score) \| 0.70 \| 0.34 \| \| 1.06 \| \| \| <0.001 \| 0.63 \| 0.21 \| \| 1.05 \| \| 0.004 \| 0.74 \| 0.39 \| \| \| \| 1.10 \| \| \| <0.001 \| \| School attendance on day of TUD (No) \| 0.21 \| -0.52 \| \| 0.94 \| \| \| 0.575 \| 0.26 \| -0.62 \| \| 1.15 \| \| 0.561 \| 0.30 \| -0.42 \| \| \| \| 1.01 \| \| \| 0.416 \| \| Season of measurement \| \| \| \| \| \| \| \| \| \| \| \| \| \| \| \| \| \| \| \| \| \| \| \| Spring \| -0.26 \| -1.12 \| \| 0.61 \| \| \| 0.565 \| -0.44 \| -1.49 \| \| 0.61 \| \| 0.409 \| -0.39 \| -1.25 \| \| \| \| 0.46 \| \| \| 0.369 \| \| Summer \| 1.10 \| -1.17 \| \| 3.37 \| \| \| 0.343 \| -2.36 \| -5.81 \| \| 1.10 \| \| 0.182 \| -0.05 \| -2.71 \| \| \| \| 2.60 \| \| \| 0.970 \| \| Autumn \| 0.17 \| -0.73 \| \| 1.06 \| \| \| 0.711 \| -0.1 \| -1.18 \| \| 0.97 \| \| 0.851 \| -0.07 \| -0.95 \| \| \| \| 0.80 \| \| \| 0.868 \| \| Multiplicative total \| -0.02 \| -0.21 \| \| 0.17 \| \| \| 0.822 \| 0.16 \| -0.07 \| \| 0.38 \| \| 0.171 \| 0.08 \| -0.10 \| \| \| \| 0.27 \| \| \| 0.387 \| \| PedsQL outcome at 10-11y \| 0.64 \| 0.58 \| \| 0.70 \| \| \| <0.001 \| 0.67 \| 0.63 \| \| 0.7 \| \| <0.001 \| 0.71 \| 0.67 \| \| \| \| 0.75 \| \| \| <0.01 \|   PA = physical activity; HRQOL = health related quality of life (PedsQL); LSAC = Longitudinal Study of Australian Children; β = model coefficient; CI = confidence interval  a. The psychosocial HRQOL summary scale is a composite of social, emotional and school HRQOL  b. The total HRQOL summary scale is a composite of physical, social, emotional and school HRQOL  c. The pubertal development scale ranged from 1 (least developed) to 4 (most developed).  **Table B6B: Associations between compositional isometric log ratios at 10-11y and additional HRQOL outcomes at 12-13y, including covariates, LSAC data**   \|  \| **Social HRQOL** \| \| \| \| \| \| \| **Emotional HRQOL** \| \| \| \| \| \| **School HRQOL** \| \| \| \| \| \| \| \| \| \| --- \| --- \| --- \| --- \| --- \| --- \| --- \| --- \| --- \| --- \| --- \| --- \| --- \| --- \| --- \| --- \| --- \| --- \| --- \| --- \| --- \| --- \| --- \| \|  \| **β** \| \| **95% CI** \| \| \| ***p*** \| \| **β** \| \| **95% CI** \| \| ***p*** \| \| **β** \| \| **95% CI** \| \| \| \| ***p*** \| \| \| \| **Composition isometric log ratio** \| \| \| \| \|  \| \| \| \| \| \| \| \| \| \| \| \|  \| \| \| \| \| \| \| Non-organized PA vs remaining \| 0.09 \| -0.13 \| \| 0.31 \| \| \| 0.411 \| 0.02 \| -0.20 \| \| 0.25 \| \| 0.848 \| -0.12 \| -0.37 \| \| \| \| 0.13 \| \| \| 0.343 \| \| Organized PA vs Active transport & Active work/chores \| 0.07 \| -0.16 \| \| 0.31 \| \| \| 0.535 \| 0.09 \| -0.14 \| \| 0.32 \| \| 0.430 \| -0.10 \| -0.36 \| \| \| \| 0.16 \| \| \| 0.459 \| \| Active transport vs Active work/chores \| 0.10 \| -0.18 \| \| 0.38 \| \| \| 0.473 \| -0.13 \| -0.40 \| \| 0.15 \| \| 0.371 \| 0.07 \| -0.23 \| \| \| \| 0.36 \| \| \| 0.669 \| \| **Covariates** \| \| \| \| \| \| \| \| \| \| \| \| \| \| \| \| \| \|  \| \| \|  \| \| \| Age (months) \| -0.03 \| -0.18 \| \| 0.11 \| \| \| 0.662 \| 0.10 \| -0.04 \| \| 0.25 \| \| 0.158 \| 0.05 \| -0.11 \| \| \| \| 0.21 \| \| \| 0.522 \| \| Sex (female) \| 0.25 \| -0.86 \| \| 1.37 \| \| \| 0.654 \| -2.03 \| -3.15 \| \| -0.92 \| \| <0.001 \| 0.74 \| -0.44 \| \| \| \| 1.93 \| \| \| 0.220 \| \| BMI (z-score) \| -0.57 \| -1.02 \| \| -0.12 \| \| \| 0.012 \| 0.47 \| -0.05 \| \| 0.99 \| \| 0.078 \| -0.33 \| -0.72 \| \| \| \| 0.06 \| \| \| 0.101 \| \| Pubertal development [a] \| -0.27 \| -1.45 \| \| 0.90 \| \| \| 0.647 \| -0.75 \| -1.96 \| \| 0.45 \| \| 0.221 \| -1.30 \| -2.58 \| \| \| \| -0.02 \| \| \| 0.047 \| \| Socioeconomic position (z-score) \| 1.02 \| 0.51 \| \| 1.52 \| \| \| <0.001 \| 0.43 \| -0.08 \| \| 0.94 \| \| 0.100 \| 1.04 \| 0.44 \| \| \| \| 1.64 \| \| \| 0.001 \| \| School attendance on day of TUD (No) \| 0.84 \| -0.19 \| \| 1.88 \| \| \| 0.111 \| -0.31 \| -1.33 \| \| 0.70 \| \| 0.547 \| -0.15 \| -1.27 \| \| \| \| 0.98 \| \| \| 0.798 \| \| Season of measurement \| \| \| \| \| \| \| \| \| \| \| \| \| \| \| \| \| \| \| \| \| \| \| \| Spring \| -0.65 \| -1.87 \| \| 0.57 \| \| \| 0.296 \| -0.14 \| -1.36 \| \| 1.07 \| \| 0.603 \| -0.46 \| -1.82 \| \| \| \| 0.90 \| \| \| 0.510 \| \| Summer \| -4.37 \| -8.66 \| \| -0.07 \| \| \| 0.047 \| -0.65 \| -4.72 \| \| 3.42 \| \| 0.754 \| -2.15 \| -7.18 \| \| \| \| 2.87 \| \| \| 0.401 \| \| Autumn \| -0.02 \| -1.28 \| \| 1.25 \| \| \| 0.977 \| -0.34 \| -1.61 \| \| 0.94 \| \| 0.603 \| -0.83 \| -2.19 \| \| \| \| 0.54 \| \| \| 0.237 \| \| Multiplicative total \| 0.14 \| -0.13 \| \| 0.40 \| \| \| 0.317 \| 0.27 \| 0.01 \| \| 0.52 \| \| 0.044 \| 0.04 \| -0.24 \| \| \| \| 0.32 \| \| \| 0.787 \| \| PedsQL outcome at 10-11y \| 0.61 \| 0.56 \| \| 0.65 \| \| \| <0.001 \| 0.61 \| 0.58 \| \| 0.64 \| \| <0.001 \| 0.42 \| 0.38 \| \| \| \| 0.47 \| \| \| <0.001 \|   PA = physical activity; HRQOL = health related quality of life (PedsQL); LSAC = Longitudinal Study of Australian Children; β = model coefficient; CI = confidence interval  a. The pubertal development scale ranged from 1 (least developed) to 4 (most developed). |
| --- | --- | --- | --- | --- | --- | --- | --- | --- | --- | --- | --- | --- | --- | --- | --- | --- | --- | --- | --- | --- | --- | --- | --- | --- | --- | --- | --- | --- | --- | --- | --- | --- | --- | --- | --- | --- | --- | --- | --- | --- | --- | --- | --- | --- | --- | --- | --- | --- | --- | --- | --- | --- | --- | --- | --- | --- | --- | --- | --- | --- | --- | --- | --- | --- | --- | --- | --- | --- | --- | --- | --- | --- | --- | --- | --- | --- | --- | --- | --- | --- | --- | --- | --- | --- | --- | --- | --- | --- | --- | --- | --- | --- | --- | --- | --- | --- | --- | --- | --- | --- | --- | --- | --- | --- | --- | --- | --- | --- | --- | --- | --- | --- | --- | --- | --- | --- | --- | --- | --- | --- | --- | --- | --- | --- | --- | --- | --- | --- | --- | --- | --- | --- | --- | --- | --- | --- | --- | --- | --- | --- | --- | --- | --- | --- | --- | --- | --- | --- | --- | --- | --- | --- | --- | --- | --- | --- | --- | --- | --- | --- | --- | --- | --- | --- | --- | --- | --- | --- | --- | --- | --- | --- | --- | --- | --- | --- | --- | --- | --- | --- | --- | --- | --- | --- | --- | --- | --- | --- | --- | --- | --- | --- | --- | --- | --- | --- | --- | --- | --- | --- | --- | --- | --- | --- | --- | --- | --- | --- | --- | --- | --- | --- | --- | --- | --- | --- | --- | --- | --- | --- | --- | --- | --- | --- | --- | --- | --- | --- | --- | --- | --- | --- | --- | --- | --- | --- | --- | --- | --- | --- | --- | --- | --- | --- | --- | --- | --- | --- | --- | --- | --- | --- | --- | --- | --- | --- | --- | --- | --- | --- | --- | --- | --- | --- | --- | --- | --- | --- | --- | --- | --- | --- | --- | --- | --- | --- | --- | --- | --- | --- | --- | --- | --- | --- | --- | --- | --- | --- | --- | --- | --- | --- | --- | --- | --- | --- | --- | --- | --- | --- | --- | --- | --- | --- | --- | --- | --- | --- | --- | --- | --- | --- | --- | --- | --- | --- | --- | --- | --- | --- | --- | --- | --- | --- | --- | --- | --- | --- | --- | --- | --- | --- | --- | --- | --- | --- | --- | --- | --- | --- | --- | --- | --- | --- | --- | --- | --- | --- | --- | --- | --- | --- | --- | --- | --- | --- | --- | --- | --- | --- | --- | --- | --- | --- | --- | --- | --- | --- | --- | --- | --- | --- | --- | --- | --- | --- | --- | --- | --- | --- | --- | --- | --- | --- | --- | --- | --- | --- | --- | --- | --- | --- | --- | --- | --- | --- | --- | --- | --- | --- | --- | --- | --- | --- | --- | --- | --- | --- | --- | --- | --- | --- | --- | --- | --- | --- | --- | --- | --- | --- | --- | --- | --- | --- | --- | --- | --- | --- | --- | --- | --- | --- | --- | --- | --- | --- | --- | --- | --- | --- | --- | --- | --- | --- | --- | --- | --- | --- | --- | --- | --- | --- | --- | --- | --- | --- | --- | --- | --- | --- | --- | --- | --- | --- | --- | --- | --- | --- | --- | --- | --- | --- | --- | --- | --- | --- | --- | --- | --- | --- | --- | --- | --- | --- | --- | --- | --- | --- | --- | --- | --- | --- | --- | --- | --- | --- | --- | --- | --- | --- | --- | --- | --- | --- | --- | --- | --- | --- | --- | --- | --- | --- | --- | --- | --- | --- | --- | --- | --- | --- | --- | --- | --- | --- | --- | --- | --- | --- | --- | --- | --- | --- | --- | --- | --- | --- | --- | --- | --- | --- | --- | --- | --- | --- | --- | --- | --- | --- | --- | --- | --- | --- | --- | --- | --- | --- | --- | --- | --- | --- | --- | --- | --- | --- | --- | --- | --- | --- | --- | --- | --- | --- | --- | --- | --- | --- | --- | --- | --- | --- | --- | --- | --- | --- | --- | --- | --- | --- | --- | --- | --- | --- | --- | --- | --- | --- | --- | --- | --- | --- | --- | --- | --- | --- | --- | --- | --- | --- | --- | --- | --- | --- | --- | --- | --- | --- | --- | --- | --- | --- | --- | --- | --- | --- | --- | --- | --- | --- | --- | --- | --- | --- | --- | --- | --- | --- | --- | --- | --- | --- | --- | --- | --- | --- | --- | --- | --- | --- | --- | --- | --- | --- | --- | --- | --- | --- | --- | --- | --- | --- | --- | --- | --- | --- | --- | --- | --- | --- | --- | --- | --- | --- | --- | --- | --- | --- | --- | --- | --- | --- | --- | --- | --- | --- | --- | --- | --- | --- | --- | --- | --- | --- | --- | --- | --- | --- | --- | --- | --- | --- | --- | --- | --- | --- | --- | --- | --- | --- | --- | --- | --- | --- | --- | --- | --- | --- | --- | --- | --- | --- | --- | --- | --- | --- | --- | --- | --- | --- | --- | --- | --- | --- | --- | --- | --- | --- | --- | --- | --- | --- | --- | --- | --- | --- | --- | --- | --- | --- | --- | --- | --- | --- | --- | --- | --- | --- | --- | --- | --- | --- | --- | --- | --- | --- | --- | --- | --- | --- | --- | --- | --- | --- | --- | --- | --- | --- | --- | --- | --- | --- | --- | --- | --- | --- | --- | --- | --- | --- | --- | --- | --- | --- | --- | --- | --- | --- | --- | --- | --- | --- | --- | --- | --- | --- | --- | --- | --- | --- | --- | --- | --- | --- | --- | --- | --- | --- | --- | --- | --- | --- | --- | --- | --- | --- | --- | --- | --- | --- | --- | --- | --- | --- | --- | --- | --- | --- | --- | --- | --- | --- | --- | --- | --- | --- | --- | --- | --- | --- | --- | --- | --- | --- | --- | --- | --- | --- | --- | --- | --- | --- | --- | --- | --- | --- | --- | --- | --- | --- | --- | --- | --- | --- | --- | --- |
