# Supplementary material for: Cross-sectional and longitudinal associations of domain-specific physical activity composition with health-related quality of life in childhood and adolescence in Australia
Source: Int J Behav Nutr Phys Act. 2023 Jun 6;20:67. doi: 10.1186/s12966-023-01466-6 (PMC10242981; doi:10.1186/s12966-023-01466-6)
Supplement: Supplementary file 3 — Additional file 3. PA domain definitions. Provides definitions of each domain of physical activity used in this study. [file 12966_2023_1466_MOESM3_ESM.pdf]

Cross-sectional and longitudinal associations of domain-specific physical activity composition with health-related quality of life in childhood and adolescence in Australia

Additional file 3

Definition of PA domains that were used in analyses (from the Longitudinal Study of Australian Children, B Cohort, 10-11y)

**Domain of PA**	**Time-use diary activities**
Organised PA	Organised athletics/gymnastics (e.g., acrobatics, athletics, cheerleading, fun runs, gymnastics, organised trampolining, sports carnivals (not further defined), track and field, triathlons)<br>Organised fitness / gym / exercise (aerobics, boot camp, calisthenics, exercise biking, exercising, fitness classes, gym classes/workouts, jogging/running, organised rope skipping)<br>Organised ball sports (various organised ball sports including baseball, basketball, bowling, cricket, football, golf/minigolf, handball, hockey, lacrosse, netball, oztag, racquet sports, rugby, soccer, softball, sports matches, t-ball, ultimate Frisbee, volleyball),<br>Organised martial arts / dancing (aikido, ballet, ballroom dancing, boxing, dancing, fencing, judo, jujitsu, karate, kickboxing, martial arts, taekwondo, wrestling)<br>Organised motor sports/roller sports/cycling (bike riding, BMX, mountain biking, roller sports, skateboarding)<br>Organised water/ice/snow Sports (bodyboarding, ice skating, kayaking, rowing, sailing, surf lifesaving, surfing, swimming, swimming carnival, water polo),<br>Organised team sports and training other (active club meetings e.g. scouts, air sport, bow hunting, flying disc games, horse riding, orienteering, rodeos, organised sports and training (other))
Non-organised PA	Non-organised athletics/gymnastics (acrobatics, cheerleading, gymnastics)<br>Non-organised fitness / gym / exercise (calisthenics, circuits, exercise biking, exercising, gym workouts, jogging, running for exercise, treadmill)<br>Non-organised ball sports (various non-organised ball sports including baseball, basketball, bowling, cricket, football, golf/minigolf, handball, hockey, lacrosse, netball, oztag, racquet sports, rugby, soccer, softball, sports matches, t-ball, ultimate Frisbee, volleyball),<br>Non-organised martial arts / dancing (boxing, wrestling, martial arts, dancing)<br>Non-organised motor sports/roller sports/cycling (bike riding, BMX, mountain biking, roller sports, skateboarding)<br>Non-organised water/ice/snow sports (bodyboarding, canoeing, ice skating, sailing, snowboarding, skiing, surf sport/surfing, swimming, water-skiing),<br>Active play (flying disc games, flying kits, hide and seek, hula hoop, kicking ball/ball games, play on play equipment, rope skipping/skipping, slip n slide, throwing ball against wall, tips/chasing/running around, trampolining, water fight)

	Other non-organised outdoor/nature PA (bush walking, exploring/going sightseeing, fishing, horse riding, unstructured archery, yabbying)
Active transport	[Travel] by bike, scooter, skateboard etc. (travel by bike/BMX, scooter, skateboard, wheelchair)<br>Travel by foot (travel by walking and running)
Active chores and work	Labourers and related workers (farm hand, labourer assistant),<br>Gardening / lawn mowing (gardening, harvesting home produce, lawn mowing, maintaining plants, outdoor /yard work, weeding, wood chopping)<br>Umpiring [paid] (umpiring)<br>Car washing [paid] (car washing),<br>Cleaning/tidying (clean bathroom/bed/bedroom/bench/floors /kitchen/laundry/ lounge room/windows, dusting, home duties/housework, mopping, move furniture, sweeping, tidying, vacuuming)<br>Cleaning grounds/garage/shed/outside of house (clean deck/garage /outside/ shed, load/unload trailer, sweep/rake leaves)<br>Pool care (chores) (clean swimming pool/pond)<br>Design/home improvement (build shed, DIY repairs, exterior/interior decoration, home renovations)<br>Walking pets/playing with pets (playing with cat/dog/pets, walking dog/pets)

For more details, see:

Kemp BJ, Parrish AM, Batterham M, Cliff DP. Changes in subdomains of non-organized physical activity between childhood and adolescence in Australia: a longitudinal study. Int J Behav Nutr Phys Act. 2022;19(1):1-11. doi: https://doi.org/10.1186/s12966-022-01311-2.
